# Dismantling the eDNA-mediated H_2_S barrier with biohybrids against refractory biofilm infections

**DOI:** 10.1016/j.bioactmat.2026.08.049

**Published:** 2026-09-15

**Authors:** Mengying Yin, Mingyue Su, Jundan Yi, Yanrong Ren, Yueting Zhang, Qian Wu, Chang Yang, Shuya Xiao, Cen Gao, Rongbing Tang, Bin Cheng, Juan Xia

**Affiliations:** a Hospital of Stomatology, Guanghua School of Stomatology, Sun Yat-sen University, Guangzhou, 510055, China; b Guangdong Provincial Key Laboratory of Stomatology, Guangzhou, 510055, China; c School of Stomatology, Lanzhou University, Lanzhou, 730000, China

**Keywords:** Biofilm-associated infections, Biohybrid, Drug delivery, Extracellular DNA, Hydrogen sulfide

## Abstract

The spatiotemporal distribution of gaseous mediators is critical for maintaining physiological functions. Biofilms both contain gas-producing bacteria and possess complex architectures that reshape gaseous mediator distribution. However, whether biofilm pathogenicity is linked to such gaseous regulation remains unclear. In this study, we reveal that extracellular DNA (eDNA) within the biofilm matrix contributes to the local retention and enrichment of H_2_S. This enrichment stabilizes eDNA, strengthens biofilm barriers, impedes gas clearance, promotes bacterial persistence, and induces macrophage immunosuppression, creating a reciprocally protective defensive loop. To dismantle this defense, we engineered a stepwise-responsive biohybrid system in which probiotic-derived engineered minicells actively target hypoxic biofilm regions, while H_2_S-triggered nanoparticle degradation enables deeper matrix penetration and concurrent H_2_S scavenging. The retained minicells then catalyze *in situ* glucose oxidation to generate H_2_O_2_, which, together with Fe^2+^-mediated Fenton-like reactions, degrades eDNA and collapses the biofilm. This strategy eradicates persistent bacteria, reverses immunosuppression, and provides a therapeutic strategy to reduce recurrence and optimize refractory infection treatments.

## Highlights

• The eDNA-rich biofilm matrix contributes to local H_2_S retention and enrichment.
• Trapped H_2_S stabilizes eDNA, drives persistence and immunosuppression.
• A stepwise biohybrid scavenges H_2_S and degrades eDNA to disrupt biofilms.
• Engineered minicells target hypoxic cores and generate H_2_O_2_ *in situ*.
• Biohybrid clears persisters, reverses immunosuppression, prevents relapse.

## Graphical abstract

**Figure:**
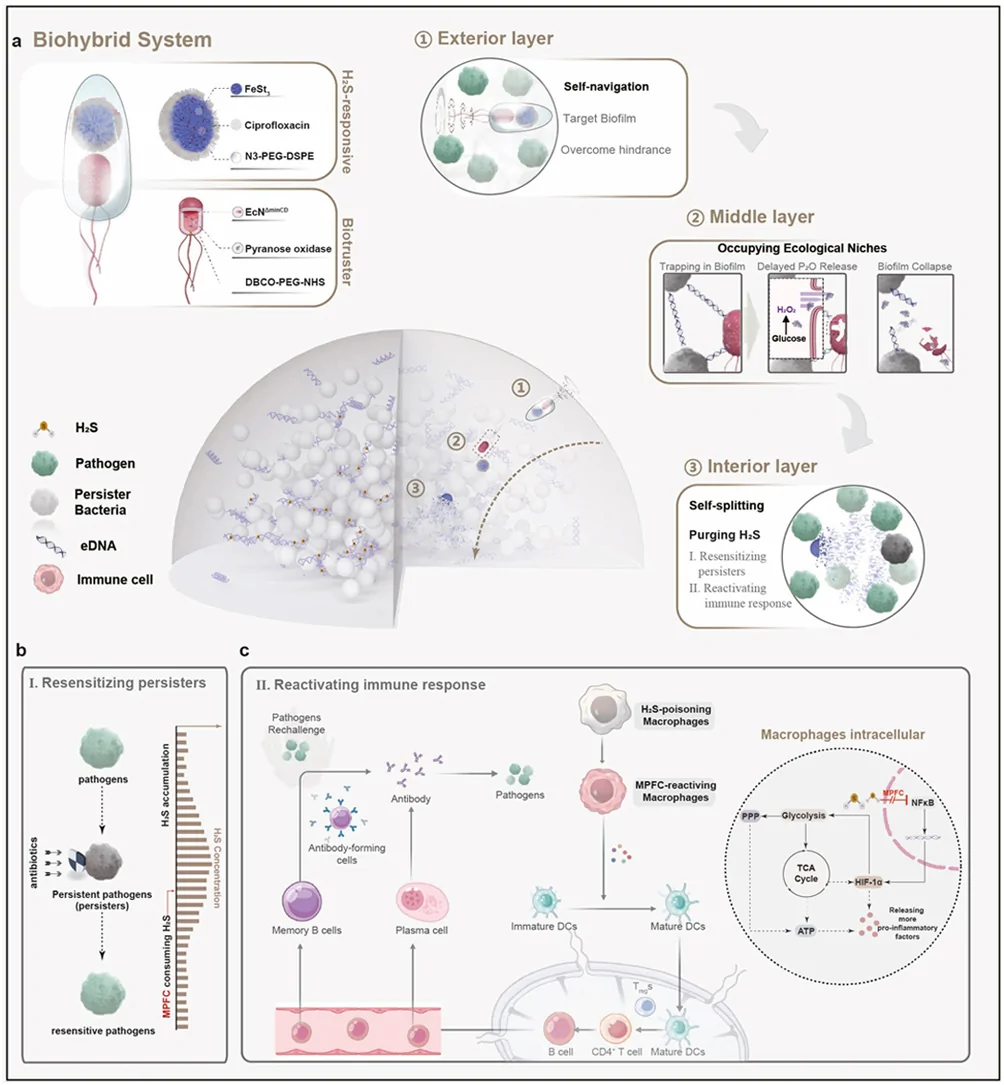

## 1 Introduction

Gaseous mediators extensively mediate cellular signal transduction, metabolic regulation, and intercellular crosstalk [[1], [2], [3]]. Their spatiotemporal distribution within living systems serves as one of the critical foundations for maintaining homeostasis [4,5]. Under normal physiological conditions, organisms maintain the ordered distribution of gaseous mediators through sophisticated structural regulation and metabolic networks. For example, the hepatic sinusoidal architecture establishes a porto-central oxygen gradient (from 60-65 mmHg to 30-35 mmHg), spatially segregating oxidative phosphorylation and glycolytic metabolism to optimize energy utilization [6]. As specialized three-dimensional microbial communities formed at host interfaces, biofilms possess complex compositions, a large specific surface area, abundant charges, and prolonged retention capacity [[7], [8], [9], [10], [11]]. In consideration of the principles of gas adsorption, these physicochemical properties of biofilms should profoundly shape gas distribution to modulate infection progress [[12], [13], [14]]. Nevertheless, the role of biofilm architecture in gas retention remains largely unexplored.

In the present study, we demonstrate that extracellular DNA (eDNA) within the extracellular polymeric substance (EPS) matrix of biofilm mediates the local retention and enrichment of hydrogen sulfide (H_2_S), establishing a distinct spatial enrichment pattern. As a key signaling molecule in the infected microenvironment, the local enrichment of H_2_S can promote bacterial persistence and suppress host immunity [[15], [16], [17]]. Notably, the biofilm skeleton-dependent spatial distribution of H_2_S creates a mutually reinforcing protective loop linking H_2_S and the biofilm matrix against anti-infective therapy. Specifically, H_2_S forms a reductive gas barrier that attenuates biofilm disruption, whereas the biofilm matrix provides spatial protection for H_2_S and hampers its targeted scavenging.

**Fig. 1:**
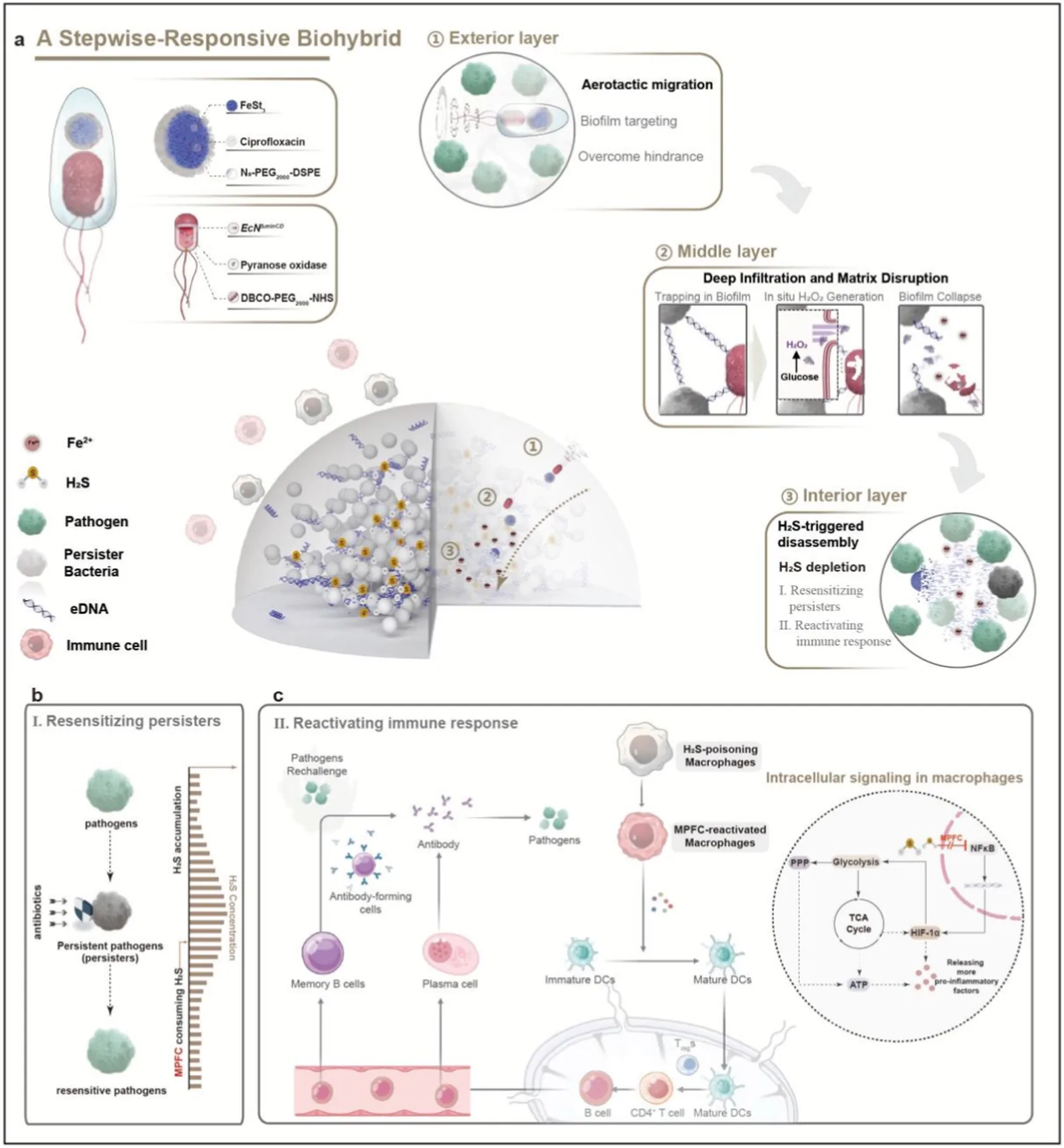
Schematic illustration of dismantling the eDNA-mediated H_2_S barrier via a stepwise biohybrid system for clearing persistent biofilm infections.

To overcome this barrier, we first engineered H_2_S-responsive iron (III) stearate/ciprofloxacin nanoparticles (FeSt_3_/Cip, denoted FC NPs) that undergo stepwise degradation in the H_2_S-rich biofilm interior. Upon exposure to the H_2_S gradient, FC NPs continuously consume H_2_S while progressively transforming into smaller secondary particles, enabling deeper penetration into the interstitial spaces of the EPS scaffold and sustained intrabiofilm antibiotic release. Through this cascade-like structural evolution, FC NPs dismantle the reductive gas barrier and weaken the protective effects of H_2_S on eDNA, bacteria, and immune cells. To enable active targeting and matrix disruption, FC NPs were anchored onto engineered minicells derived from *Escherichia coli* Nissle 1917 Δ*minCD* (*EcN*^Δ*minCD*^), yielding a stepwise biohybrid system termed minicell^P2O^@FC (MPFC) (Fig. 1). These probiotic-derived minicells constitutively express pyranose oxidase (P_2_O) and retain motility toward anaerobic environments [18,19], thereby facilitating biohybrid transport toward the oxygen-depleted biofilm interior. After FC-mediated consumption of H_2_S, the minicells further infiltrate the loosened biofilm and catalyze *in situ* glucose oxidation to continuously generate H_2_O_2_, which triggers Fenton-like degradation of eDNA and ultimately drives collapse of the biofilm architecture. In summary, this strategy effectively eradicates persisters and reverses the immunosuppressive phenotype of macrophages, thereby providing a therapeutic strategy for reducing infection recurrence and improving the treatment of refractory biofilm-associated infections.

## 2 Results and discussion

### 2.1 eDNA mediates H_2_S enrichment within the biofilm matrix

**Fig. 2:**
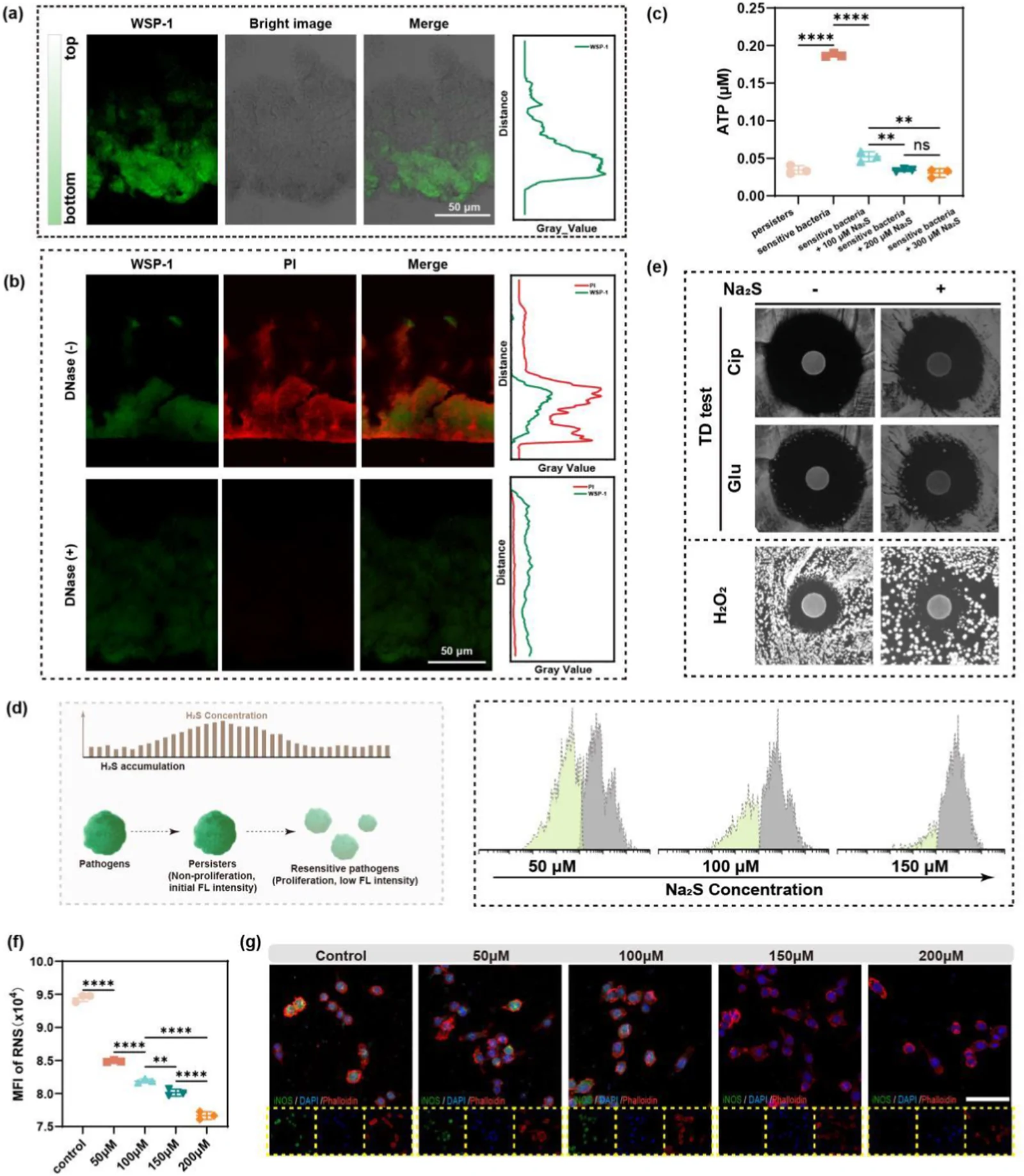
Extracellular DNA mediates H_2_S enrichment within the biofilm matrix and contributes to a pathological protective microenvironment. (a) Representative confocal images of cryosectioned biofilms stained with the H_2_S probe WSP-1, together with the corresponding depth-dependent fluorescence intensity profile. (b) Representative fluorescence images of *S. aureus* biofilms stained with WSP-1 (H_2_S, green) and propidium iodide (PI; eDNA, red) in the absence or presence of DNase I pretreatment, together with the corresponding fluorescence intensity line-scan profiles. (c) Intracellular ATP levels in persisters and sensitive bacteria following treatment with different concentrations of Na_2_S. (d) Schematic illustration of the fluorescence dilution (FD) assay used to distinguish growing cells with diluted fluorescence from non-growing persister cells with retained high fluorescence (top), along with representative flow cytometry histograms showing the proportion of non-growing (persister) *S. aureus* after treatment with different concentrations of Na_2_S (bottom). (e) TDtest and H_2_O_2_ inhibition zone assays showing that Na_2_S exposure promotes bacterial persistence and reduces susceptibility to oxidative stress. Glu: glucose. (f) Mean fluorescence intensity (MFI) of reactive nitrogen species (RNS) in RAW264.7 macrophages treated with different concentrations of Na_2_S. (g) Representative immunofluorescence images of iNOS expression in macrophages after different treatment. Nuclei were stained with DAPI (blue), and F-actin was stained with rhodamine phalloidin (red). Scale bar  =  50 μm.

To characterize the spatial distribution of H_2_S within the biofilm microenvironment, mature biofilms were cryosectioned and stained with the H_2_S fluorescent probe WSP-1. Confocal imaging revealed pronounced vertical spatial heterogeneity in H_2_S distribution, with fluorescence intensity markedly enriched in the deeper regions of the biofilm, whereas signals in the superficial layers remained comparatively weak (Fig. 2a). Notably, this gaseous distribution pattern spatially correlated with the structural gradient of the EPS matrix, suggesting that the dense EPS scaffold may promote local H_2_S retention through combined physicochemical interactions and structural confinement. Among the major EPS components, eDNA serves as a key structural scaffold that maintains the compact architecture of the deep biofilm layers and is widely recognized as an important therapeutic target for disrupting recalcitrant biofilms [20,21]. We therefore hypothesized that eDNA, while providing mechanical support to the biofilm, also contributes to deep-layer H_2_S retention and enrichment. To test this hypothesis, eDNA in biofilms was labeled with propidium iodide (PI) and compared with the distribution of H_2_S. Notably, the regions with high H_2_S intensity overlapped well with PI-positive eDNA-rich domains, supporting a close spatial association between H_2_S and eDNA (Fig. 2b). We then functionally perturbed eDNA using deoxyribonuclease (DNase) pretreatment. Following DNase-mediated degradation of eDNA, the intrabiofilm H_2_S signal became more homogeneous with no obvious enrichment (Fig. 2b), supporting an important contribution of eDNA to the formation and maintenance of localized H_2_S-enriched regions within the biofilm.

To further evaluate this retention effect, equal amounts of intact and DNase-treated lyophilized biofilms were incubated with Na_2_S solution for 24 h, and the residual H_2_S concentration in the supernatant was measured using an H_2_S microelectrode. The residual H_2_S concentration was significantly lower in the intact biofilm group than in the DNase-treated group, demonstrating greater H_2_S retention by the eDNA-containing biofilm matrix (Fig. S1). Molecular dynamics simulations further revealed a mild preferential distribution of H_2_S near the hydrated DNA phosphate backbone, although the contacts were transient and non-site-specific (Fig. S2). Together, these findings support a contribution of eDNA to local H_2_S retention within the biofilm matrix.

Direct microelectrode measurements further quantified the extent of this enrichment. The H_2_S concentration within the intact biofilm interior reached 705.95 ± 24.28 μM, which was significantly higher than that in the corresponding supernatant (295.26 ± 25.05 μM), providing direct quantitative evidence of local H_2_S enrichment within the biofilm (Fig. S3). Following DNase treatment, the intrabiofilm H_2_S concentration decreased to 548.58 ± 33.79 μM, corresponding to a reduction of approximately 22.3% relative to the intact biofilm. Although the H_2_S concentration in the DNase-treated biofilm remained higher than that in the supernatant, its significant decrease after eDNA disruption further supports the contribution of the eDNA-containing matrix to local H_2_S retention.

This deep H_2_S reservoir does not merely reflect passive accumulation of metabolic by-products, but is also likely to have pathological consequences. Elevated H_2_S levels suppressed intracellular ATP production (Fig. 2c) and attenuated bacterial proliferation (Fig. 2d), thereby driving bacteria residing in the deep biofilm layers into a dormant persister state with drastically reduced susceptibility to metabolism-dependent antibiotics (Fig. 2e) [22]. Beyond its protective role for bacteria, H_2_S accumulation within the infected microenvironment also compromised host immune surveillance. Because direct microelectrode measurements placed the H_2_S concentration in the biofilm supernatant at approximately 300 μM, graded Na_2_S concentrations of 50-200 μM were used in the homogeneous macrophage culture system to model controlled bulk-phase sulfide exposure. These concentrations were not overtly cytotoxic to macrophages but were sufficient to suppress inducible nitric oxide synthase (iNOS) expression and consequently reduce reactive nitrogen species (RNS) production, thereby impairing macrophage bactericidal activity (Fig. 2f and g).

This eDNA-dependent spatial distribution establishes a mutually protective interplay between H_2_S and the biofilm matrix. Na_2_S pretreatment significantly attenuated H_2_O_2_-mediated degradation of extracted biofilm eDNA (Fig. S4), indicating that H_2_S protects eDNA against oxidative damage. Conversely, the eDNA-rich matrix contributes to local H_2_S retention, supporting a reciprocal protective relationship between H_2_S and the biofilm matrix.

### 2.2 Construction and characterization of MPFC

**Fig. 3:**
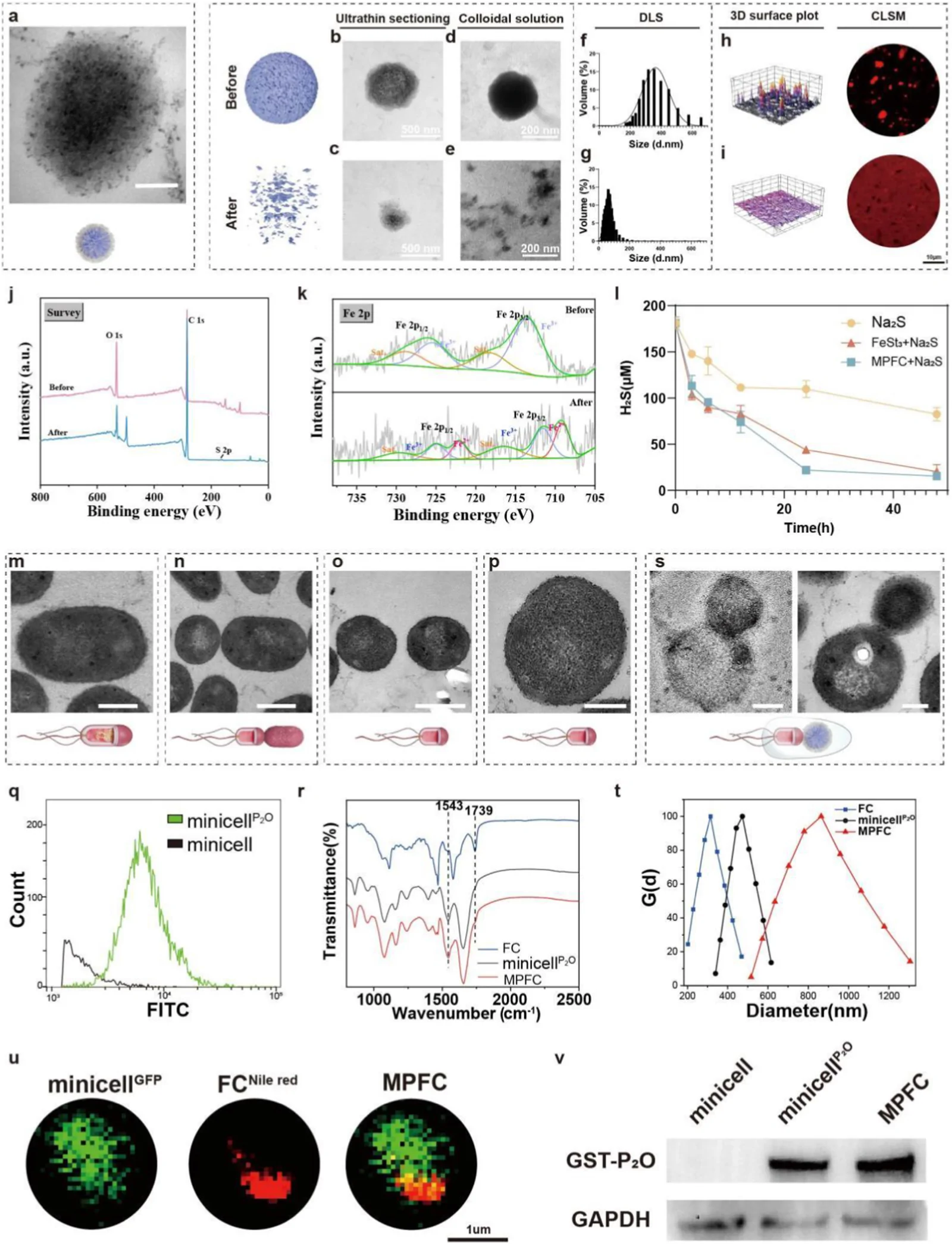
Synthesis and characterization of MPFC. (a) TEM image of FeSt_3_/Cip nanoparticles (FC NP). Scale bar = 100 nm. (b–e) TEM images showing the fragmentation of FC NPs before and after treatment with Na_2_S. (f-g) Hydrodynamic diameter of FC NPs before and after Na_2_S treatment. (h-i) 3D surface plots of CLSM images visualizing the fluorescence intensity distribution of Nile Red within FC NPs before and after H_2_S-induced disintegration. (j) Full XPS survey spectra of FC NPs before and after Na_2_S treatment. (k) High-resolution Fe 2p XPS spectra showing the increase in the Fe(II) content after H_2_S treatment. (l) Time-dependent depletion of dissolved H_2_S by FeSt_3_ NPs and MPFC in comparison with the Na_2_S control. (m) TEM image of the parent strain, *Escherichia coli* Nissle 1917 (*EcN*). Scale bar = 500 nm. (n-p) TEM images of the engineered Δ*minCD* strain (*EcN*^Δ*minCD*^) and the purified, nonproliferating minicells derived from it. (p) Magnified view of the boxed region in (o). Scale bars = 500 nm (n, o) and 200 nm (p). (q) Flow cytometry analysis confirming EGFP expression in P_2_O-expressing minicells (minicell^P2O^) but not in control minicells. (r) FTIR spectra of FC NPs, minicell^P2O^, and the final MPFC biohybrid. (s) Representative TEM image of MPFC, showing FC NPs anchored on the surface of a minicell. Scale bar = 200 nm. (t) Hydrodynamic diameter distributions of FC NPs, minicell^P2O^, and MPFC measured by DLS. (u) CLSM images showing the association between Nile Red-labeled FC NPs (red) and EGFP-expressing minicells (green) in MPFC. Scale bar = 1 μm. (v) Western blot analysis confirming the expression of the GST-P_2_O fusion protein in minicell^P2O^ and MPFC.

Motivated by the identification of this reciprocally protective defensive loop formed by eDNA and H_2_S, we engineered a stepwise-responsive biohybrid system designed to penetrate and dismantle this protective barrier. The fabrication strategy is schematically illustrated in Fig. S5. We first focused on constructing the H_2_S-responsive particulate module. To this end, iron(III) stearate (FeSt_3_) and ciprofloxacin (Cip) were co-assembled via an emulsification method to generate FC NPs, which serve as both H_2_S scavengers and antibiotic reservoirs. Transmission electron microscopy (TEM) imaging showed that the resulting FC NPs were spherical and uniform, with an average diameter of ∼400 nm (Fig. 3a). UV-vis spectrophotometry further revealed a high Cip loading content of 59.43% (Fig. S6), indicating substantial drug payload capacity.

A key design feature of FC NPs is their H_2_S-triggered structural transformation. We hypothesized that endogenous H_2_S within the biofilm interior would reduce Fe^3+^ in the nanocomplexes to Fe^2+^, thereby disrupting the FeSt_3_-based particle skeleton and initiating on-demand decomposition. To test this, FC NPs were incubated in Na_2_S solution to mimic the H_2_S-rich biofilm microenvironment. Within 10 min, TEM imaging revealed pronounced structural collapse, with the initially compact FC NPs fragmenting into ultrasmall and heterogeneous secondary particles (Fig. 3b–e). DLS confirmed the H_2_S-triggered size change. Across three independent batches, the untreated FC NPs showed reproducible size distributions, with an average diameter of 381.93 ± 3.93 nm and a PDI of 0.192 ± 0.042; after Na_2_S treatment, the diameter consistently decreased to 60.59 ± 1.99 nm (Fig. 3f–g). Concomitantly, confocal laser scanning microscopy (CLSM) imaging visualized rapid payload diffusion, as evidenced by the dispersion of the initially concentrated red fluorescence (Fig. 3h–i). These results demonstrate that FC NPs can undergo stepwise disassembly and size reduction in response to H_2_S, a property expected to facilitate deeper penetration into the EPS interstices while enabling sustained intrabiofilm drug release. To clarify the chemical basis of this transformation, we performed X-ray photoelectron spectroscopy (XPS). After reaction with Na_2_S, a distinct sulfur signal appeared in the survey spectrum (Fig. 3j), and high-resolution Fe 2p spectra showed an increase in Fe(II) species to 48.68% (Fig. 3k), consistent with H_2_S-induced iron reduction and iron sulfide formation. Functionally, this reaction led to efficient depletion of dissolved H_2_S over 48 h (Fig. 3l), which was further corroborated by lead acetate strip assays (Fig. S7). Together, these data establish FeSt_3_ NPs as a morphologically transformable and chemically reactive module that simultaneously scavenges H_2_S and releases antibiotics in a biofilm-relevant microenvironment.

After establishing the particulate module, we next introduced a biological carrier to further improve transport and catalytic matrix disruption. Using the CRISPR-Cas9 system, we deleted the *minCD* gene from the genome of *Escherichia coli* Nissle 1917 (*EcN*) [18,19], thereby disrupting normal binary fission and generating achromosomal, non-proliferating minicells with a uniform diameter of approximately 500 nm (Fig. 3m–p). To endow these minicells with oxidative activity, a plasmid encoding P_2_O (pGEX-4T-P2O-T2A-EGFP) was transformed into the parental *EcN*^Δ*minCD*^ strain by electroporation (Fig. S8). The resulting engineered minicells, hereafter termed minicell^P2O^, were purified by sequential antibiotic selection and density gradient centrifugation. Flow cytometry and CLSM confirmed successful retention of the plasmid-encoded EGFP signal, indicating inheritance of the engineered traits from the parental strain (Fig. 3q and S9).

To integrate the two modules, FC NPs were first functionalized with azide groups using DSPE-PEG_2000_-N_3_, whereas the surface of minicell^P2O^ was modified with DBCO-PEG_2000_-NHS. The two components were then covalently conjugated through strain-promoted alkyne-azide cycloaddition (SPAAC) click chemistry to generate the final biohybrid [23], termed MPFC. The nomenclature and composition of the different constructs are summarized in Supplementary Table S1. Fourier transform infrared (FTIR) spectroscopy confirmed successful integration by revealing characteristic peaks derived from both FC NPs and minicells in the MPFC group (Fig. 3r). TEM directly visualized FC NPs anchored to the surface of minicells, demonstrating formation of a stable hybrid architecture (Fig. 3s). Further TEM analysis showed that the predominant morphology was a single FC nanoparticle associated with a single minicell. When smaller FC nanoparticles were used, multiple nanoparticles could attach to one minicell (Fig. S10), suggesting that steric constraints may favor the observed 1:1 assembly pattern under the current formulation. DLS further supported this assembly, showing an increase in hydrodynamic diameter to approximately 850 nm, which was clearly distinct from either FC NPs alone or bare minicells (Fig. 3t). CLSM imaging also confirmed this structure, with Nile Red-labeled FC NPs (red) localized on EGFP-expressing minicells (green) (Fig. 3u).

Importantly, the assembled MPFC retained the H_2_S-responsive behavior and H_2_S-scavenging capability of FC NPs, indicating that biohybrid formation did not compromise the reactive functionality of the particulate module (Fig. 3l and S7). Meanwhile, the engineering process preserved the biological functionality of the minicell carrier. In addition to the retained endogenous esterase activity (Fig. S11), we further examined whether *EcN*-derived minicells retained any intrinsic antimicrobial or antibiofilm activity associated with the parental strain (Fig. S12) [[24], [25], [26]]. In addition, Western blot analysis using an anti-GST antibody confirmed the expression of the GST-P_2_O fusion protein in both minicell^P2O^ and MPFC lysates, whereas no corresponding band was detected in control minicells (Fig. 3v). Finally, the assembled MPFC system exhibited good biocompatibility, as demonstrated by negligible cytotoxicity toward mammalian cells in Cell Counting Kit-8 (CCK-8) assays (Fig. S13).

### 2.3 MPFC actively targets and penetrates biofilms

**Fig. 4:**
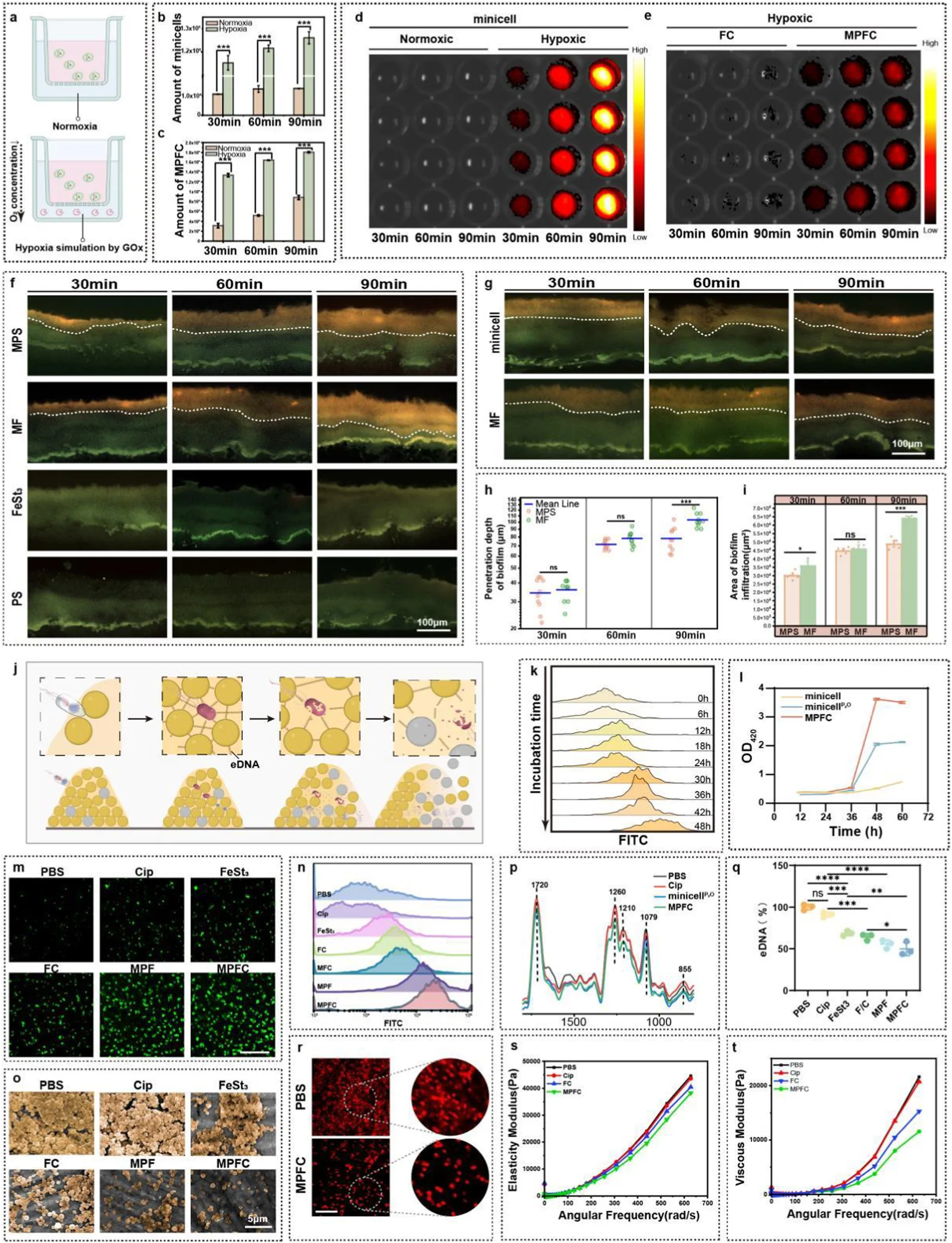
MPFC targets and penetrates biofilms. (a) Schematic illustration of the experimental design used to evaluate the chemotactic migration of MPFC toward hypoxic environments. (b–c) Quantification of the chemotactic migration ability of minicell^GFP^ and MPFC^GFP^ through flow cytometry. (n = 3). (d) Fluorescence imaging of GFP-labeled minicells in the lower chamber of a Transwell system after 30, 60, and 90 min of incubation under normoxic or hypoxic conditions (n = 4). (e) Fluorescence imaging of Nile Red-labeled FC and MPFC in the lower chamber of a Transwell system after 30, 60, and 90 min of incubation under hypoxic conditions (n = 4). (f) CLSM images of FITC-ConA-labeled *S. aureus* biofilm cross-sections after treatment with four different red fluorescent particles: PS (polystyrene), FeSt_3_ NP, MPS (minicell@PS), and MF (minicell@FeSt_3_). Scale bar = 100 μm. (g) Trajectory tracking of mCherry-expressing minicells (minicell^mCherry^ or MF^mCherry^) within FITC-ConA-labeled biofilm. Scale bar = 100 μm. (h-i) Quantitative analysis of the penetration depth (h) and area (i) of the dispersed fluorescence signal calculated from CLSM images using ImageJ. (j) Schematic illustration of niche occupation by minicell^P2O^ and the subsequent production of H_2_O_2_ via P_2_O, resulting in eDNA degradation. (k) Time-dependent intracellular ROS accumulation in minicells, as measured by DCFH-DA. (l) Quantitative analysis of H_2_O_2_ release from minicell^P2O^ and MPFC formulations at different incubation times. (m–n) CLSM imaging (m) and flow cytometry (n) analysis of ROS levels in *S. aureus* cells after treatment with various formulations. MPF: minicell^P2O^@FeSt_3_. Scale bar = 20 μm. (o) Morphological changes in *S. aureus* biofilms after different treatments. Scale bar = 5 μm. (p) ATR-FTIR spectra of biofilms subjected to different treatments. (q) Quantification of total eDNA content in biofilm matrices after treatment, measured using a NanoDrop 2000c spectrophotometer (n = 3). (r) Representative PI-stained images showing eDNA degradation within biofilms in the PBS and MPFC groups. Scale bar = 100 μm. (s–t) Rheological characterization of biofilm mechanical properties. Measurement of the elastic modulus (s) and viscous modulus (t) of biofilms after different treatments. Frequency sweeps were performed at 1% strain.

As the propulsion unit of MPFC, minicell^P2O^ leverages the rotary motors of its flagella to autonomously explore and propel MPFC towards the biofilm along the oxygen concentration gradient. To evaluate hypoxia-directed migration, we established a Transwell system in which oxygen in the lower chamber was depleted using a GOx/catalase oxygen-scavenging system (Fig. 4a) [27,28]. The oxygen-sensitive probe [Ru(dpp)_3_]Cl_2_ confirmed markedly reduced oxygen availability in the GOx-treated lower chamber compared with the normoxic control (Fig. S14). Importantly, direct comparison between the two Transwell compartments further showed greater oxygen depletion in the lower chamber than in the upper chamber under hypoxic conditions (Fig. S14), confirming the establishment of a vertical oxygen gradient. Minicell-containing formulations preferentially migrated toward the hypoxic lower chamber (Fig. 4b–e). In contrast, paraformaldehyde-fixed minicells showed an approximately 88.5% reduction in lower-chamber accumulation compared with live minicells (Fig. S14), supporting a predominantly motility-dependent migration process.

Upon reaching the biofilm via chemotaxis, the biohybrid progressively infiltrated the intricate internal architecture of the matrix. By tracking the trajectories of minicells (mCherry, red) within biofilms labeled with FITC-ConA (green), we demonstrated that the minicells actively moved and eventually localized within the central regions of the biofilm, regardless of whether they carried a payload (Fig. 4g). This finding indicates that deep infiltration is an intrinsic capability of the minicell-based motility system. When the incubation time was extended to 90  min, the red fluorescence from the minicell@FeSt_3_ (MF) group spread throughout the biofilm, whereas that from minicell@PS (MPS) remained confined to the middle region (Fig. 4f, h, and i). Consistently, three-dimensional reconstruction of complete CLSM z-stacks showed a broader particle distribution throughout the biofilm in the MF group than in the PS, FeSt_3_, and MPS groups (Fig. S15). This dispersal suggests that the FeSt_3_ NPs in the biohybrid undergo a redox reaction with dissolved H_2_S. With the progression of the reaction, the particles gradually disintegrate into smaller secondary particles, thereby overcoming the spatial constraints imposed by the biofilm matrix. Compared with non-deformable polystyrene (PS) microspheres of similar dimensions, the H_2_S-responsive particles exhibited broader distribution within the biofilm, thereby improving intrabiofilm delivery. Consistent with this, MPFC infiltration led to a pronounced attenuation of intrabiofilm H_2_S signals (Fig. S16), thereby dismantling the reductive gas barrier and, in turn, sensitizing the matrix to H_2_O_2_/ROS-driven oxidative degradation.

After completing its delivery mission, minicell^P2O^ was trapped in the framework of the biofilm, where it could continue cellular processes such as metabolism and protein production. Owing to the absence of chromosomal DNA, the minicells were insensitive to Cip. Accordingly, the minicells remained viable within the biofilm during MPFC treatment. The minicell^P2O^ continuously generated H_2_O_2_ through P_2_O-mediated glucose oxidation (Fig. 4j). Rather than being permanently retained intracellularly, H_2_O_2_ underwent dynamic production and membrane efflux. Intracellular oxidative stress increased at earlier time points (Fig. 4k), whereas extracellular H_2_O_2_ became more pronounced at 36-48  h (Fig. 4l), coinciding with progressive membrane damage. We hypothesized that the reaction between FeSt_3_ and H_2_S results in the formation of metastable iron sulfides, which subsequently release catalytically active ferrous ions (Fe^2+^) into the local microenvironment. These released Fe^2+^ ions then catalyze the decomposition of H_2_O_2_ into highly reactive hydroxyl radicals (•OH) via a Fenton-like reaction (Fig. 4m and n), accelerating biofilm collapse (Fig. 4o). To confirm that this efficient ROS generation was indeed mediated by the release of Fe^2+^, we introduced the iron chelator ethylenediaminetetraacetic acid (EDTA) and found that it significantly suppressed ROS production (Fig. S17), confirming that the catalytic process is dependent on the ferrous ions generated as a by-product of the reaction. These results complete the designed reaction cascade, in which FeSt_3_ links sulfide consumption and particle disassembly to Fe^2+^-dependent Fenton-like ROS amplification.

To further elucidate the mechanism underlying biofilm collapse, we analyzed biofilms after different treatments using attenuated total reflection Fourier-transform infrared (ATR-FTIR) spectroscopy [29]. The results revealed a marked loss of nucleic acid-associated signals in the MPFC-treated group (Fig. 4p), consistent with the quantitative DNA measurements (Fig. 4q). *In situ* fluorescent staining with PI verified that the change in nucleic acid density was attributable to the degradation of eDNA rather than intracellular DNA (Fig. 4r). As a determinant for the stabilization of the biofilm, a lack of eDNA caused the elastic modulus (Fig. 4s) and viscous modulus (Fig. 4t) of the biofilm to decrease sharply [30,31], which accounted for the MPFC-induced dispersion of the biofilm.

### 2.4 MPFC attenuates persistence-associated tolerance within biofilms

**Fig. 5:**
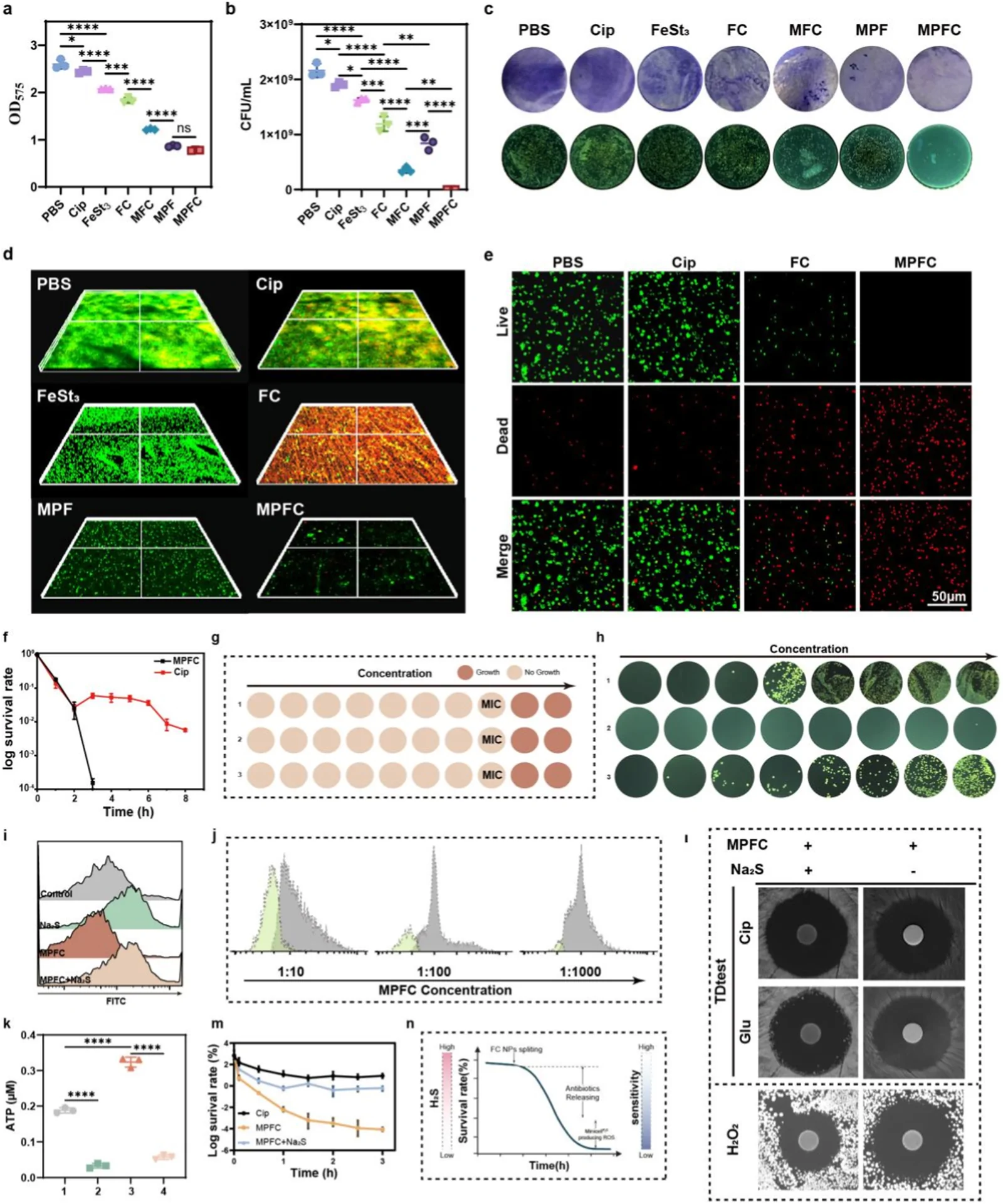
MPFC attenuates persistence-associated tolerance within biofilms. (a) Quantification of the remaining biofilm biomass by crystal violet staining. (b) Bacterial survival counts within biofilms determined by CFU plating. (c) Representative macroscopic images of crystal violet-stained biofilms formed on titanium discs and the corresponding colony growth on agar plates after different treatments. (d-e) Representative live/dead CLSM images of mature *S. aureus* biofilms after different treatments. Live bacteria are stained green; dead bacteria are stained red. (f) Time‒kill kinetics of Cip and MPFC against bacteria isolated from mature biofilms. (g) Minimum inhibitory concentration (MIC) and (h) minimum bactericidal concentration (MBC) of Cip and MPFC against planktonic *S. aureus* with or without an exogenous H_2_S donor (Na_2_S) (1:Cip, 2:MPFC, 3:MPFC + Na_2_S). (i) Intracellular H_2_S levels in bacteria after treatment with different formulations. (j) Representative flow cytometry histograms of fluorescence dilution experiments tracking the proportion of non-growing (persisters; gray) *S. aureus* and sensitive bacteria (green) when treating with different concentration of MPFC. (k) Intracellular ATP levels in bacteria after different treatments (1: Control, 2: Na_2_S, 3: MPFC, 4: MPFC + Na_2_S). (l) Representative TDtest images showing suppression of antibiotic persistence by MPFC pretreatment (top) and enhanced susceptibility to exogenous H_2_O_2_ after MPFC-mediated H_2_S depletion (bottom). (m) Time-kill kinetics of biofilm-derived *S. aureus* after treatment with different formulations. (n) Schematic illustration of the temporal separation between early H_2_S depletion and subsequent H_2_O_2_ release during MPFC treatment.

Encouraged by the confirmed H_2_S-scavenging and eDNA-dismantling capabilities of MPFC, we next systematically evaluated its efficacy against persister bacteria embedded within mature *S. aureus* biofilms. As shown in Fig. 5a–d, staining and quantitative plate-counting assays collectively revealed a marked reduction in bacterial viability following MPFC treatment, with only sparse viable bacteria detected within the disrupted biofilm architecture. Notably, the majority of *S. aureus* dispersed into the supernatant following MPFC treatment were effectively eliminated (Fig. 5e), and the density of viable *S. aureus* in the MPFC-treated group was reduced to merely 0.11% of that in the Cip-treated group (Fig. 5b). In stark contrast, Cip treatment prolonged the minimum duration for killing (MDK) and generated a characteristic biphasic killing curve, whereas MPFC treatment abolished this biphasic pattern entirely (Fig. 5f). This observation indicates that a subpopulation of *S. aureus* remained viable upon exposure to lethal antibiotic concentrations, a phenotype consistent with bacterial persistence rather than acquired resistance [[32], [33], [34]].

To delineate the mechanistic origin of this refractory subpopulation, we further characterized planktonic *S. aureus* isolated from biofilms treated with different formulations. The minimum inhibitory concentration (MIC) of Cip remained unchanged across all treatment groups, whereas the minimum bactericidal concentration (MBC) was significantly higher in the Cip-treated group than in the MPFC-treated counterparts (Fig. 5g–h). The divergence between MBC and MIC, rather than an elevated MIC, is a defining criterion that distinguishes bacterial persistence from acquired resistance, thereby confirming the persistent phenotype of the surviving subpopulation [[32], [33], [34]]. Critically, supplementation with excess Na_2_S markedly attenuated the bactericidal efficacy of MPFC (Fig. 5h), implicating intrabiofilm H_2_S as the primary protective mediator and suggesting that MPFC exerts its anti-persister activity principally through H_2_S scavenging.

To further validate this hypothesis mechanistically, we quantified intracellular H_2_S levels and persister abundance as a function of MPFC concentration. MPFC treatment significantly reduced intracellular H_2_S levels (Fig. 5i), and the abundance of persister cells showed a corresponding inverse dose-dependent relationship with MPFC concentration (Fig. 5j) [35]. Furthermore, intracellular ATP levels in the MPFC-treated group were elevated approximately 2-fold relative to untreated controls (Fig. 5k), supporting metabolic reactivation following H_2_S depletion. Beyond persister resensitization (Fig. 5l–m), H_2_S depletion by MPFC further potentiated ROS-mediated bactericidal activity. Notably, kinetic measurements showed that H_2_S depletion by FeSt_3_-containing formulations occurred during the early treatment phase (Fig. 3l), whereas intracellular ROS accumulation and appreciable H_2_O_2_ release from P_2_O-engineered minicells occurred later (Fig. 4k–l). To evaluate the functional relevance of this temporal sequence, we further compared different treatment orders. FeSt_3_ pretreatment followed by H_2_O_2_ exposure resulted in significantly greater bacterial killing than H_2_O_2_ alone or the reverse H_2_O_2_-to-FeSt_3_ sequence (Fig. S18). Together, these findings indicate that the temporal separation between early H_2_S depletion and later H_2_O_2_ release may therefore provide a window for persisters to recover metabolic activity before oxidative stress, thereby increasing their susceptibility to subsequent ROS-mediated killing (Fig. 5n).

### 2.5 MPFC reprograms macrophage metabolism to enhance immune responses

To assess whether MPFC could reverse this H_2_S-induced immunosuppression, we established an *in vitro* coculture model in which macrophages were exposed to *S. aureus* biofilms (Fig. S19). To distinguish the therapeutic effect of MPFC from any intrinsic immunogenicity, we first evaluated the direct effects of its individual components on macrophages in the absence of biofilm. Control experiments showed that minicells exhibited limited intrinsic immunostimulatory activity at the dose used in this study. The complete MPFC system elicited only a mild increase in IL-1β levels, with no significant changes in TNF-α or IL-6 levels (Fig. S20).

**Fig. 6:**
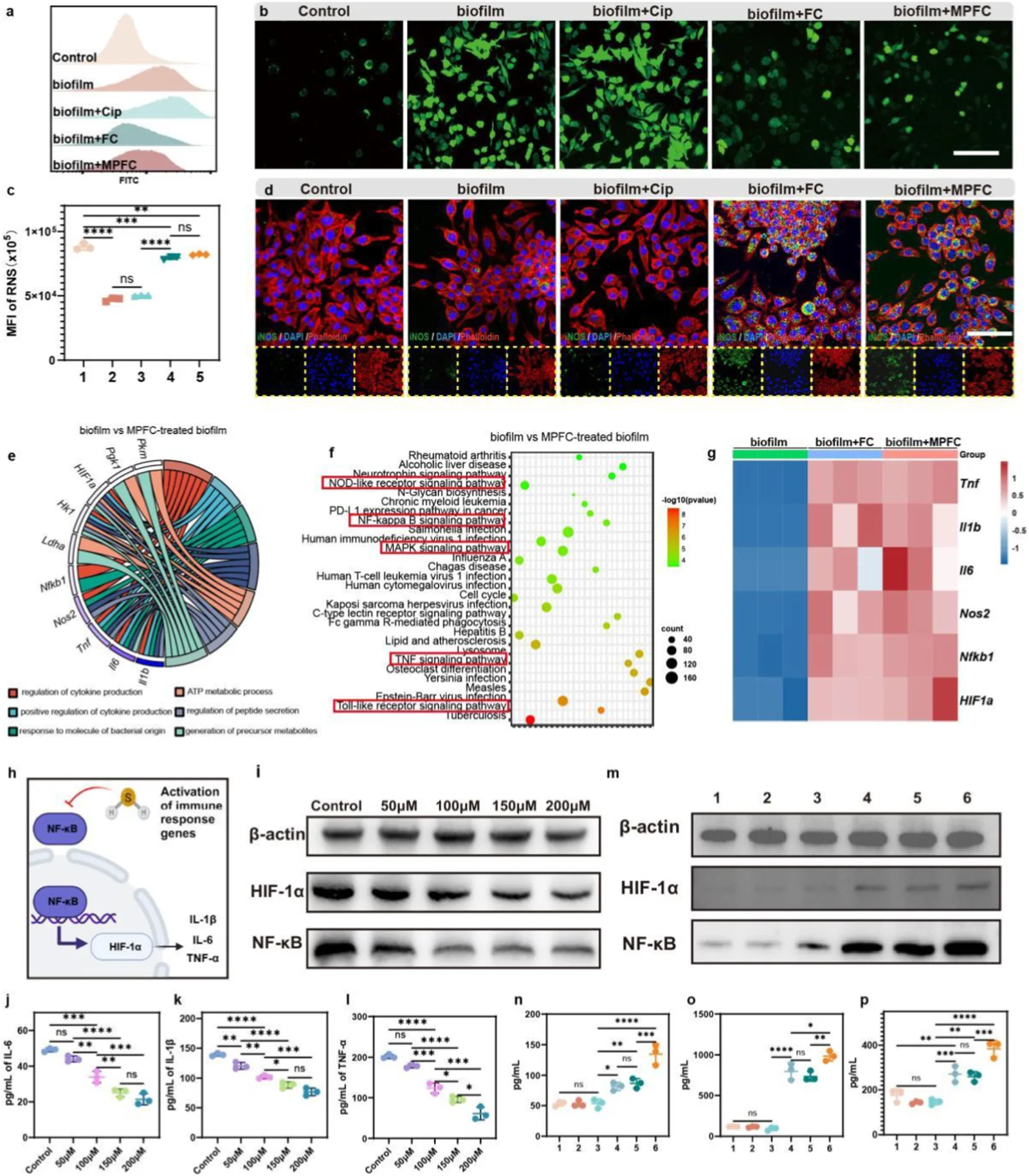
MPFC reverses H_2_S-induced macrophage immunosuppression by activating the NF-κB–HIF-1α signaling axis. (a–b) Intracellular H_2_S levels in macrophages cocultured with biofilms after various treatments were determined by a WSP-1 probe via (a) flow cytometry and (b) CLSM. Scale bar = 20 μm. (c) MFI of RNS in macrophages after different treatments in the coculture system (1: control, 2: biofilm, 3: biofilm + Cip, 4: biofilm + FC, 5: biofilm + MPFC). (d) Representative immunofluorescence images showing restored iNOS expression in macrophages after FC and MPFC treatment. Scale bar = 50 μm. (e) Chord diagram of Gene Ontology (GO) pathway enrichment analysis and a corresponding heatmap of key differentially expressed genes. (f) Top 30 enriched KEGG pathways from transcriptomic analysis. (g–h) Heatmaps of differentially expressed genes within the NF-κB-HIF-1α pathway (g) and a schematic illustration of this signaling axis (h). (i) Western blot analysis of NF-κB and HIF-1α in macrophages treated with Na_2_S. (j-l) Effects of different treatments on the NF-κB/HIF-1α pathway and downstream cytokine production in macrophages. (m) Representative Western blot showing the protein levels of NF-κB and HIF-1α. (n-p) Concentrations of secreted proinflammatory cytokines, including (n) IL-6, (o) IL-1β, and (p) TNF-α, were measured by ELISA. Treatment groups: Control (1, untreated macrophages); biofilm (2, macrophages cocultured with PBS-treated biofilm); biofilm + Cip/FeSt_3_/FC/MPFC (3–6, macrophages cocultured with biofilms treated with Cip, FeSt_3_, FC, or MPFC, respectively).

In the biofilm coculture model, MPFC treatment significantly lowered intracellular H_2_S levels in macrophages (Fig. 6a–b). Given the high membrane permeability of H_2_S, depletion of biofilm-associated extracellular H_2_S by the FC-containing formulations (Fig. S16) would reduce H_2_S availability for diffusion into adjacent macrophages. Thus, the decreased intracellular H_2_S signal is primarily attributed to extracellular H_2_S depletion rather than direct intracellular scavenging by FC NPs. This reduced H_2_S exposure was accompanied by recovery of iNOS expression and RNS production in the FC and MPFC groups (Fig. 6c–d). To elucidate the underlying mechanism, we performed transcriptomic and metabolomic analyses on macrophages from different treatment groups (biofilm, biofilm + FC, biofilm + MPFC). Gene Ontology (GO) and Kyoto Encyclopedia of Genes and Genomes (KEGG) enrichment analyses revealed that the differentially expressed genes were significantly enriched in pathways related to immune responses, cellular metabolism, and pathogen recognition (Fig. 6e–f). These analyses highlighted NF-κB-HIF-1α-associated signaling as a potential regulatory link between H_2_S depletion and macrophage immunometabolic reactivation (Fig. 6g–h). Previous work has shown that excessive H_2_S can inhibit NF-κB, thereby suppressing the transcription of its downstream target, HIF-1α, a master regulator of cellular adaptation to stress [[36], [37], [38]]. Consistent with this, we observed that H_2_S suppressed NF-κB and HIF-1α expression (Fig. 6i–l), whereas MPFC treatment restored their expression by mitigating intracellular H_2_S accumulation (Fig. 6m–p). To further establish the functional requirement of HIF-1α, we performed siRNA-mediated knockdown of HIF-1α in macrophages (Fig. S21). This knockdown significantly abrogated the biofilm + MPFC-induced upregulation of proinflammatory cytokines (IL-1β, IL-6 and TNF-α), providing direct evidence that HIF-1α is an indispensable mediator of MPFC-driven immune enhancement (Fig. S22). To further strengthen the causal relationship, we performed a genetic rescue experiment by re-expressing siRNA-resistant HIF-1α in HIF-1α-knockdown macrophages. Restoration of HIF-1α expression significantly rescued the cytokine response to MPFC treatment (Fig. S23).

**Fig. 7:**
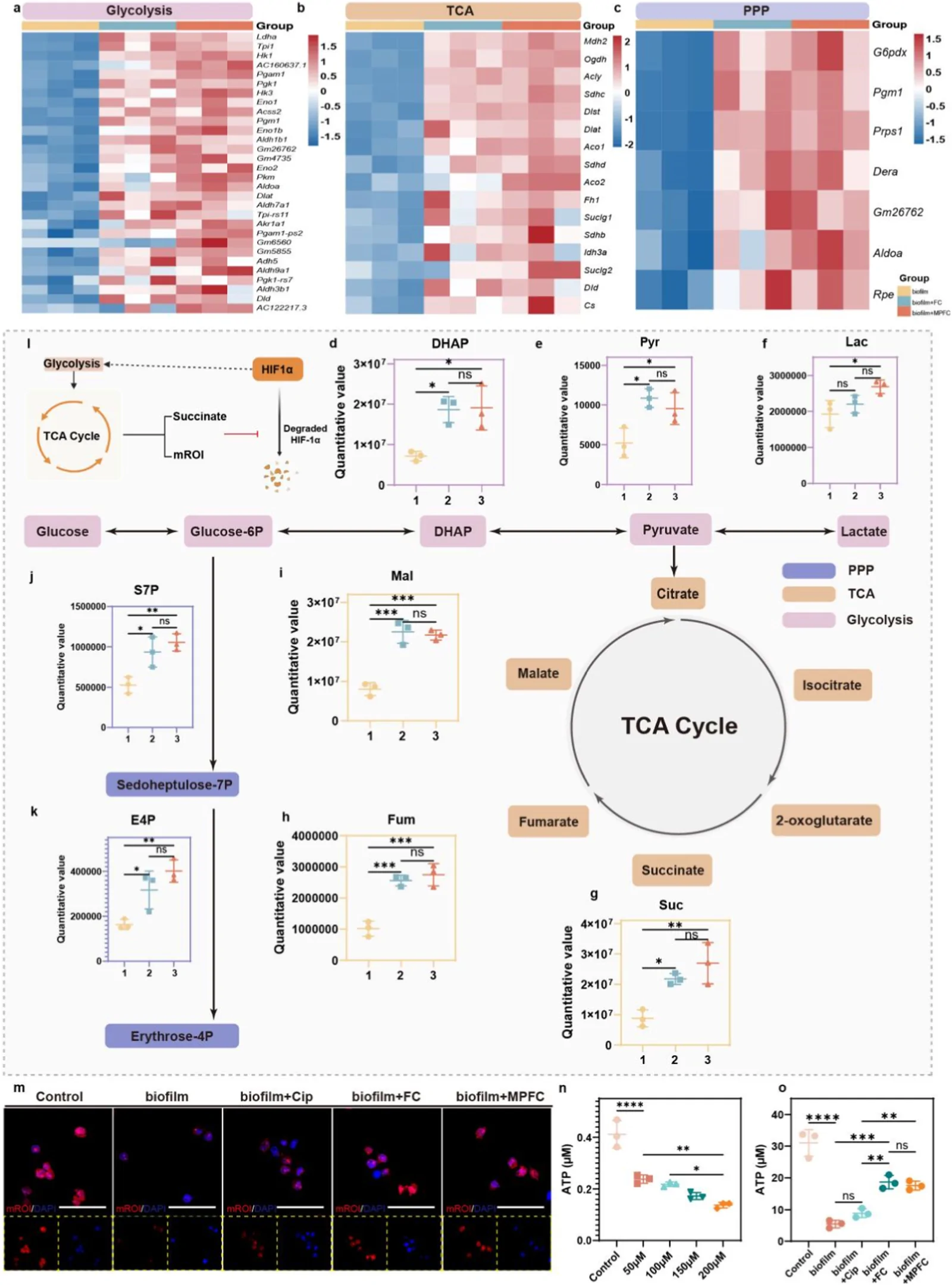
MPFC promotes central carbon metabolism by eliminating H_2_S. (a-c) Heatmaps from RNA-seq data showing the differential expression of genes involved in (a) glycolysis, (b) the tricarboxylic acid (TCA) cycle, and (c) the pentose phosphate pathway (PPP) in macrophages after the indicated treatments (n = 3). (d–k) Normalized levels of key metabolites measured by metabolomics. Levels of glycolytic intermediates: (d) dihydroxyacetone phosphate (DHAP), (e) pyruvate (Pyr), and (f) lactate (Lac). Levels of TCA cycle intermediates: (g) succinate (Suc), (h) fumarate (Fum), and (i) malate (Mal). Levels of PPP intermediates: (j) sedoheptulose-7-phosphate (S7P) and (k) erythrose-4-phosphate (E4P). (l) Schematic diagram illustrating a positive feedback loop in which TCA-derived succinate and mitochondrial reactive oxygen intermediates (mROIs) stabilize HIF-1α by inhibiting prolyl hydroxylases (PHDs), thereby promoting further glycolysis and succinate accumulation. (m) Representative fluorescence images of the mROI (red) detected by the MitoSOX Red probe in macrophages after different treatments. Nuclei were counterstained with DAPI (blue). Scale bar = 30 μm. (n) Cellular adenosine triphosphate (ATP) levels in macrophages treated with different concentrations of the H_2_S donor Na_2_S. (o) Restoration of cellular ATP levels in macrophages after different treatments in the biofilm coculture model.

Consistent with the functional involvement of HIF-1α in glycolytic regulation, MPFC treatment was accompanied by broad remodeling of central carbon metabolism. HIF-1α is known to upregulate glycolytic enzymes [39], and our transcriptomic data confirmed the upregulation of *Pgk1*, *Hk1*, *Ldha*, and *Pkm* in the MPFC-treated group (Fig. 7a). This increase in glycolytic flux provides metabolic intermediates that fuel both the pentose phosphate pathway (PPP) and the tricarboxylic acid (TCA) cycle (Fig. 7a–c). Metabolomic analysis confirmed a broad increase in intermediates involved in glycolysis (Fig. 7d–f), the TCA cycle (Fig. 7g–i), and the PPP (Fig. 7j–k) following MPFC treatment. The attenuation of MPFC-induced lactate accumulation following HIF-1α knockdown further supports a functional contribution of HIF-1α to the glycolytic component of this metabolic response (Fig. S24).

To confirm that this metabolic shift is functionally required for the observed immune enhancement, we treated macrophages with the glycolysis inhibitor 2-deoxy-D-glucose (2-DG). The inhibition of glycolysis with 2-DG markedly attenuated the MPFC-induced proinflammatory response, confirming that the metabolic increase and immune activation are mechanistically coupled (Fig. S25). Notably, a residual increase in IL-1β expression after MPFC treatment persists even with glycolysis inhibition, which may be attributable to the intrinsic mild immunostimulatory properties of the MPFC (Fig. S20).

This metabolic reprogramming also initiates a positive feedback loop that stabilizes HIF-1α [40,41]. The accumulation of the TCA cycle intermediate succinate, which was observed in our MPFC-treated group (Fig. 7g), is known to stabilize HIF-1α by inhibiting prolyl hydroxylases (PHDs). Concurrently, an increase in mitochondrial reactive oxygen intermediates (mROIs), another byproduct of the revitalized TCA cycle, also contributed to PHD inhibition and HIF-1α stabilization (Fig. 7l–m and S26). This combined metabolic enhancement ultimately restored the cellular ATP levels, which had previously been depleted by H_2_S, to provide the necessary energy for robust phagocytic and secretory functions in macrophages (Fig. 7n–o).

### 2.6 MPFC treats and prevents infection *in vivo*

**Fig. 8:**
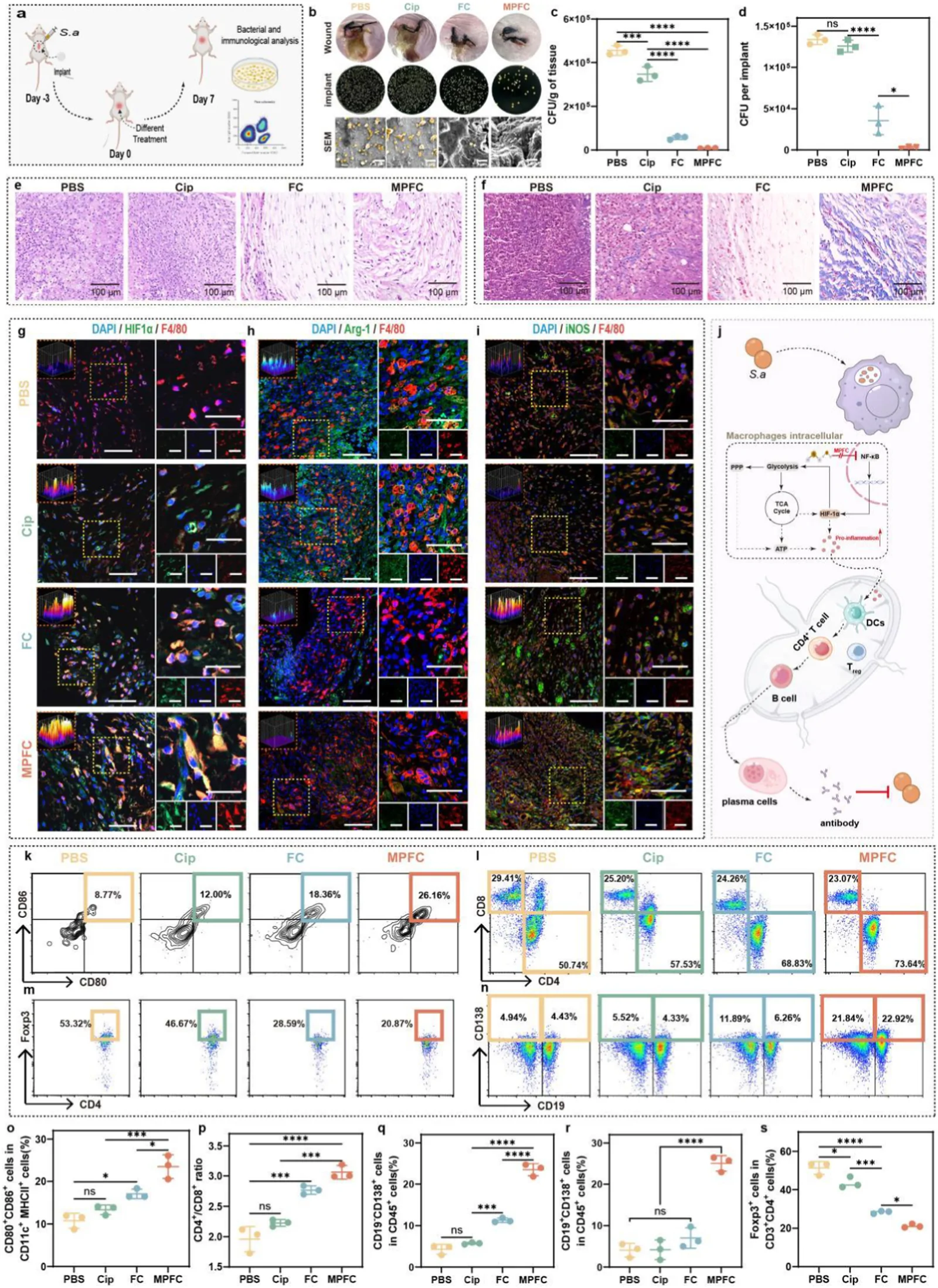
MPFC enhances antibiofilm humoral immunity *in vivo*. (a) Schematic illustration of the subcutaneous *S. aureus* implant infection model and the therapeutic regimen. (b) Representative images of the wound area, bacterial load on explanted titanium plates, and SEM images of the *in vivo* biofilm structure after treatment. (c–d) Quantification of bacterial CFU counts in peri-implant tissues (c) and on explanted implants (d) from infected mice. (e–f) Representative H&E staining (e) and Masson's staining (f) of tissues from the infected area. Scale bar = 100 μm. (g–i) Immunofluorescence staining images and corresponding 3D fluorescence distribution maps of the tissues surrounding the implants. Stains: F4/80 for macrophages (red); DAPI for nuclei (blue); and the specific markers HIF-1α, Arg-1, or iNOS (green). Scale bar = 100 μm. (j) Schematic illustration of MPFC-stimulated antibiofilm humoral immunity. (k–n) Representative flow cytometry plots of cells isolated from the inguinal draining lymph nodes (IDLNs) showing (k) mature DCs (CD80^+^CD86^+^) gated on CD11c^+^MHC-II^+^ cells, (l) CD4^+^ T cells (CD4^+^) gated on CD3^+^ T cells, (m) Tregs (Foxp3^+^) gated on CD3^+^CD4^+^ T cells, and (n) plasmablasts (CD138^+^CD19^+^), plasma cells (CD138^+^CD19^−^) gated on CD45^+^ cells. (o–s) Quantitative analysis of the corresponding immune cell populations.

To assess the therapeutic potential of the MPFC system in a physiologically relevant context, we conducted *in vivo* studies utilizing a subcutaneous implant-associated infection model, which closely mimics the challenging microenvironment of chronic biofilm infections (Fig. 8a). To further evaluate biofilm targeting *in vivo*, Nile Red-labeled MPFC or FC was administered distal to the infected implants. After 24 h, MPFC showed markedly greater accumulation at the biofilm-covered implant surface than FC, supporting enhanced *in vivo* biofilm localization mediated by the minicell module (Fig. S27). In *S. aureus*-infected mice, MPFC treatment significantly accelerated wound healing, with treated areas showing markedly diminished redness and swelling (Fig. 8b). This clinical improvement was underpinned by a profound bactericidal effect, as MPFC reduced the bacterial burden to 8.2 × 10^3^ CFU/g in peripheral tissue and 2.9 × 10^3^ CFU per implant, representing a multilog reduction (Fig. 8c–d). To further assess bacterial persistence *in vivo*, viable bacteria recovered from peri-implant tissues were subjected to the TDtest. Late-emerging colonies were markedly reduced after FC treatment and were nearly absent in the MPFC group, indicating a substantial reduction in the viable persister population *in vivo* (Fig. S28). Histological analysis further supported these findings. Haematoxylin and eosin (HE) and Masson's trichrome staining of peri-implant tissues revealed that MPFC treatment promoted robust tissue repair, characterized by minimal neutrophil infiltration and substantial, well-organized collagen deposition (Fig. 8e–f).

In agreement with our *in vitro* results, MPFC treatment effectively remodelled the local immune microenvironment. Immunofluorescence staining revealed that macrophages (F4/80^+^) in the MPFC-treated group exhibited a proinflammatory, bactericidal phenotype, as evidenced by the significant upregulation of HIF-1α and iNOS and the downregulation of the M2-like marker arginase-1 (Arg-1) (Fig. 8g–i). To quantitatively assess the local macrophage response, single-cell suspensions isolated from peri-implant tissues were analyzed by flow cytometry. MPFC treatment significantly increased the proportion of CD86^+^ macrophages and the ratio of CD86^+^ to CD206^+^ macrophages compared with the PBS and Cip groups, whereas CD206^+^ macrophages showed a nonsignificant decreasing trend (Fig. S29). These results broadly supported the macrophage phenotypic changes observed by immunofluorescence. This local immunoactivation elicited a robust, systemic adaptive immune response within the infection-draining lymph nodes (IDLNs) (Fig. 8j). Immune profiling of the IDLNs demonstrated that MPFC activated multiple immune cell populations involved in humoral immunity. The proportion of mature dendritic cells (DCs) (CD11c^+^MHC-II^+^CD80^+^CD86^+^) increased to 26.16% (Fig. 8k–o). Consistent with these *in vivo* findings, *in vitro* flow cytometry showed that biofilm exposure suppressed DC maturation, whereas MPFC markedly restored the CD80^+^CD86^+^ DC population (Fig. S31). Mature DCs undergo antigen processing and secrete abundant proinflammatory cytokines including TNF-α, IL-1β, and IL-6, to modulate the immune system of lymph cells. As expected, a significantly greater number of CD4^+^ T cells and a sharp decrease in T_reg_ cells (immunosuppressive cells) were observed in the MPFC group (Fig. 8l–m, p and s). In the absence of the restriction of T_reg_ cells, CD4^+^ T cells can selectively activate B cells to increase the number of plasma cells (CD138^+^CD19^−^) and plasmablasts (CD138^+^CD19^+^), thereby supporting antibody production as part of the systemic humoral response (Fig. 8n–q-r).

**Fig. 9:**
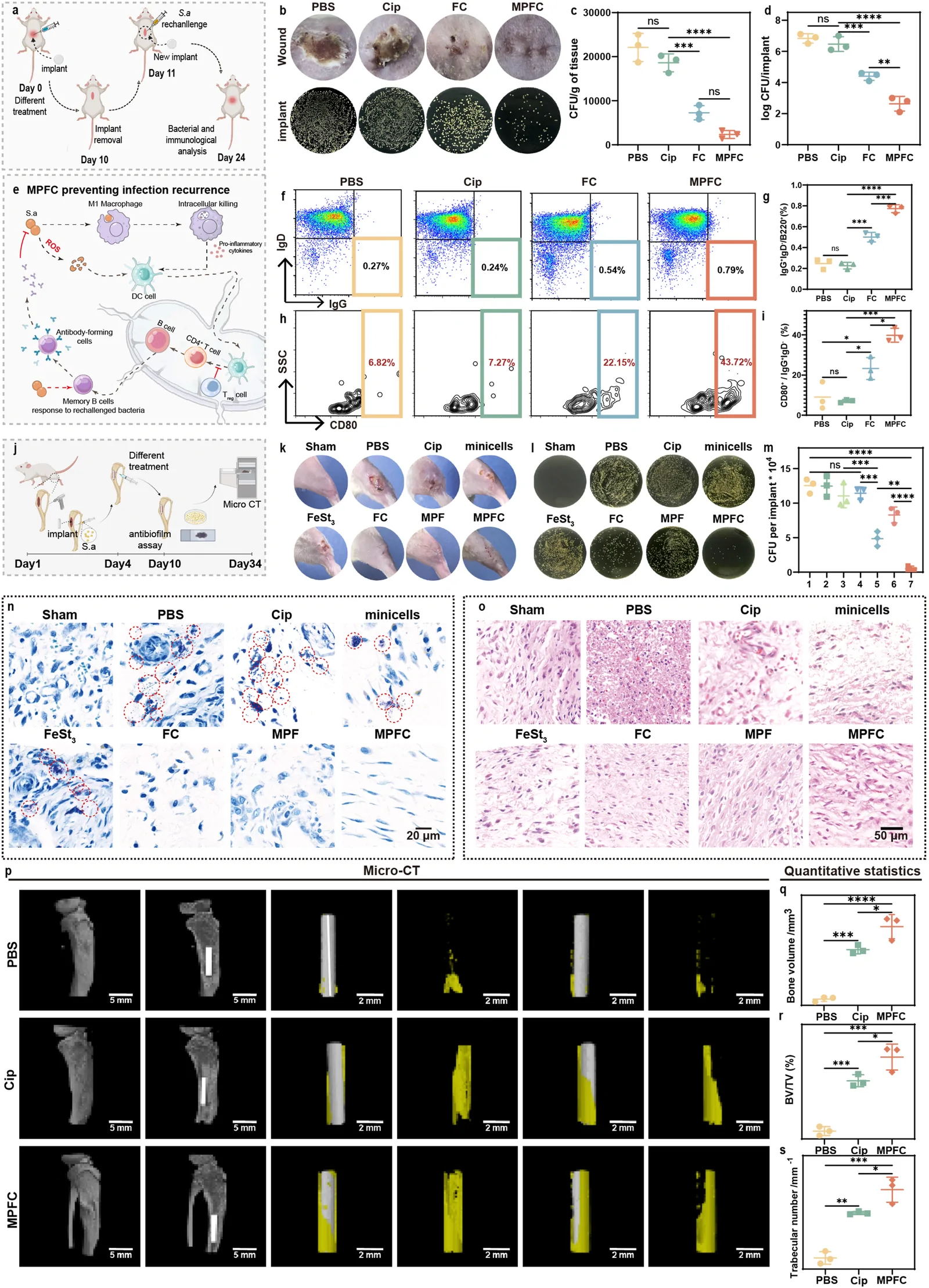
MPFC prevents recurrent infections and treats intraosseous implant infections. (a) Schematic illustration of the establishment and experimental procedure for the mouse model of recurrent implant infection. (b) Representative photographs of infectious areas and bacterial loads on explanted titanium plates from mice after the indicated treatments in the primary infection phase. (c-d) Quantification of bacterial CFU counts in peri-implant tissues (c) and on explanted implants (d) after bacterial rechallenge in the recurrent infection phase. (e) Schematic illustrating immune cell interactions upon bacterial rechallenge. (f-g) Representative flow cytometry plots (f) and statistical analysis (g) of memory B cells (Bmem, IgG^+^IgD^−^) gated on B220^+^ cells. (h-i) Representative flow cytometry plots (h) and quantitative analysis (i) of CD80^+^ Bmem cells gated on IgG^+^IgD^−^ cells. (j) Schematic illustration of the establishment and experimental procedure for the rat model of intraosseous implant infection. (k) Representative photographs of the infected areas. (l) Bacterial load on explanted titanium rods. (m) Quantification of bacterial CFU counts from peri-implant bone tissue. (1: PBS, 2: Cip, 3: minicells, 4: FeSt_3_, 5: FC, 6: MPF, 7: MPFC). (n-o) Giemsa staining (n) and H&E staining (o) of peri-implant tissues. *S. aureus* colonies are indicated by circles. (p) Representative micro-CT images of infected rat tibiae after the indicated treatments. (q-s) Quantitative statistics of the bone volume (BV) (q), bone volume/total volume ratio (BV/TV) (r), and trabecular number (Tb.N) (s) calculated from the micro-CT data.

To determine whether MPFC could prevent infection recurrence, we established a model of recurrent implant-related infection (IRI) [42]. After Ti plate replacement surgery, the mice in the IRI model were rechallenged with *S. aureus* (Fig. 9a). Although antibiotic treatment alleviated the symptoms of the primary infection, almost all of the mice experienced recurrent infection. As expected, MPFC treatment drastically inhibited primary infection without causing infection relapse (Fig. 9b). Fourteen days after the second inoculation of the pathogen, the *S. aureus* burden in MPFC-treated mice was negligible in the peripheral tissue and significantly decreased on the Ti plate (Fig. 9c and d). Furthermore, as shown in Fig. S35, MPFC treatment resulted in the lowest infiltration of neutrophils and pathogens, as well as increased collagen deposition. Together, these findings demonstrate that MPFC markedly improved resistance to bacterial rechallenge.

To determine whether this improved resistance was accompanied by a humoral memory-associated response, we harvested the IDLNs and further characterized the corresponding B-cell populations. As shown in Fig. 9f–g, MPFC treatment resulted in the highest frequency of class-switched memory-phenotype B cells (Bmem, IgG^+^IgD^−^; Fig. S36). Notably, the proportion of CD80^+^ Bmem cells, which are capable of rapidly differentiating into antibody-forming cells upon antigen re-exposure, was also significantly increased in the MPFC group (Fig. 9h–i). In parallel, total serum IgG levels were significantly higher in MPFC-treated group than in the PBS, Cip, and FC groups (Fig. S37). Together, this results support a systemic humoral memory-associated response following MPFC treatment and its association with improved resistance to bacterial rechallenge.

To further evaluate the therapeutic potential of MPFC in a more clinically challenging setting, we employed a rat model of intraosseous implant infection (Fig. 9j). Compared with other groups, clinical symptoms were obviously mitigated (Fig. 9k) and bacterial burden was sharply reduced in MPFC group (Fig. 9l–n). Histological analysis confirmed superior tissue healing and bacterial clearance (Fig. 9n and o). Most importantly, MPFC treatment effectively promoted osseointegration. Microcomputed tomography (micro-CT) imaging and quantitative analysis revealed that MPFC markedly suppressed infection-associated bone resorption and facilitated the formation of substantial new bone on the implant surface, as evidenced by significantly greater bone volume (BV), bone volume/total volume ratio (BV/TV), and trabecular number (Tb.N) (Fig. 9p–s). Finally, the H&E staining of major organs revealed no signs of toxicity, confirming the excellent biosafety of the MPFC system *in vivo* (Fig. S38).

## 3 Conclusion

This study demonstrates that the pathogenicity of biofilms stems not only from their role as physical barriers to drug penetration, but also from their capacity to promote local H_2_S retention through the eDNA-rich biofilm matrix, thereby establishing a protective microenvironment that supports infection persistence. The abnormal enrichment of H_2_S within mature biofilm regions further stabilizes the eDNA-based scaffold, reinforces bacterial persistence, and induces local immunosuppression, creating a mutually reinforcing protective interaction that supports infection persistence. These findings expand the current view of chronic biofilm infection beyond structural defense by demonstrating that biofilm architecture also regulates the local retention and distribution of H_2_S.

Guided by this eDNA-dependent H_2_S retention mechanism, we engineered a stepwise-responsive biohybrid system (MPFC) that dismantles this protective barrier through coordinated H_2_S scavenging, eDNA scaffold degradation, and infected-microenvironment remodeling, thereby simultaneously relieving bacterial persistence and immune suppression and enabling effective treatment of refractory biofilm infections.

More broadly, our work raises the possibility that the abnormal retention and spatial redistribution of gaseous mediators may contribute to the persistence of certain diseased microenvironments. Whether analogous structure-dependent regulation of gaseous mediators occurs beyond biofilm-associated infections, for example, in oral mucosal inflammation, intestinal dysbiosis, or the tumor microenvironment, warrants further investigation. The therapeutic potential of H_2_S regulation has also been demonstrated in other pathological settings [43]. Recent advances in biomimetic nanoplatforms, probiotic-derived engineered carriers, and multifunctional nanoparticle systems further illustrate the potential of integrating biological interfaces with functional materials for targeted delivery and microenvironment modulation [[44], [45], [46]]. Accordingly, investigating structure-dependent gaseous-mediator retention may identify additional therapeutic opportunities for chronic refractory infections and other microenvironment-associated diseases.

## 4 Materials and methods

**Materials.** DBCO-PEG_2000_-NHS and DSPE-PEG_2000_-N_3_ were obtained from Shanghai Ponsure Biotech, Inc. Iron(III) stearate (FeSt_3_) was obtained from TCI (Shanghai) Development Co., Ltd. Nile red, sodium sulfide (Na_2_S) and NaHS·xH_2_O were obtained from Aladdin Biochemical Technology Co. (Shanghai, China). All NaHS and Na_2_S solutions were freshly prepared and used immediately to maintain concentration stability. Ciprofloxacin (Cip) and ampicillin (Amp) were obtained from Macklin Biochemical Technology Co. (Shanghai, China). L-α-Lecithin was obtained from Beijing Solarbio Science & Technology Co., Ltd. The titanium plates and titanium rods were from Shengdaxing Metal Materials Co. (Baoji, China). FITC-ConA was obtained from Shanghai Maokang Biotechnology Co., Ltd.

**Bacteria, Cells and Animals.** *Staphylococcus aureus* (*S. aureus*, ATCC 25923) was obtained from the American Type Culture Collection. Individual colonies of *S. aureus* were transferred to lysogeny broth (LB) medium in a shaking incubator at 37 °C overnight. The bacteria were then harvested and washed three times with PBS. The precipitate obtained after centrifugation was used immediately for further experiments.

RAW264.7 cells were purchased from Hunan Fenghui Biotechnology Co., Ltd. (Changsha, China). Following recovery from liquid nitrogen, cells were incubated at 37 °C under 5% CO_2_ atmosphere in incubator. High-glucose Dulbecco's modified Eagle's medium (DMEM) (Hyclone, Logan, UT, USA), 10% fetal bovine serum (ExCell Bio, Shanghai, China), and 1% penicillin/streptomycin (Wuhan Procell Biotechnology Co., Ltd.) were used to culture RAW264.7 cells. The medium was replaced every 2 days.

BALB/c mice and SD rats were purchased from Animal Experimental Center, Lanzhou University and Animal Experimental Center, Sun Yat-sen University.

**Biofilm Cryosectioning and Imaging.** Sterilized and air-dried polycarbonate membranes were aseptically placed on the surface of LB agar plates. Exponential-phase *S. aureus* was inoculated onto the center of each membrane and incubated at 37 °C until a visibly raised mature biofilm formed at the membrane center. For eDNA degradation experiments, mature biofilms were treated with DNase I (Solarbio, China) before harvesting, while untreated biofilms served as controls.

For spatial analysis of intrabiofilm signals, the biofilms were carefully collected and embedded in optimal cutting temperature (OCT) compound. The embedded samples were frozen and longitudinally sectioned using a cryostat. The resulting sections were stained with the H_2_S-responsive fluorescent probe WSP-1 (Maokangbio, MX5301) or propidium iodide (PI, Beyotime, Shanghai, China), as required by the experimental design. After staining, the sections were gently rinsed with PBS to remove excess dye and immediately subjected to confocal laser scanning microscopy (CLSM) to examine the fluorescence distribution within the biofilm.

**H_2_S retention assay using lyophilized biofilms.** Mature *S. aureus* biofilms were prepared as described above and divided into untreated and DNase-treated groups. After treatment, the biofilms were collected and lyophilized. Equal amounts of lyophilized biofilm from each group were incubated with Na_2_S solution for 24 h. Na_2_S solution without biofilm was included as a control. After incubation, the residual H_2_S concentration in the supernatant was quantified using an H_2_S-selective microelectrode (Unisense). Sulfide retention by the biofilm matrix was evaluated based on the decrease in residual H_2_S concentration relative to the biofilm-free control.

**Molecular dynamics simulation.** The NMR structure of a 28-bp duplex DNA (PDB ID: 2M2C) was used as a representative eDNA model. The DNA was solvated in a physiological aqueous environment containing 0.15 M NaCl using the Solution Builder module of CHARMM-GUI, generating an 80 × 80 × 80 Å^3^ periodic box. One H_2_S molecule was subsequently introduced into the simulation box using Packmol to complete the atomic model of the system. The system was then parameterized using AmberTools25. DNA was described using the OL24 force field, water using the OPC four-point model, and monovalent ions using parameters compatible with the OPC water model. H_2_S parameters were independently generated using Antechamber with the GAFF2 force field and AM1-BCC partial charges, with missing parameters completed using Parmchk2. The resulting parameters were combined using LEaP to generate the AMBER topology and coordinate files. After energy minimization, the system was gradually heated to 310 K and equilibrated for 5 ns under the NPT ensemble, followed by a 300-ns conventional molecular dynamics simulation using GPU-accelerated AMBER24. The resulting trajectories were analyzed using cpptraj to calculate radial distribution functions of H_2_S relative to DNA phosphate and nucleobase atoms and to characterize transient H_2_S-DNA contacts and residence behavior.

**Microelectrode measurement of intrabiofilm H_2_S.** Mature *S. aureus* biofilms were prepared and either left untreated or treated with DNase as described above. H_2_S concentrations were measured using an H_2_S-selective microelectrode after calibration according to the manufacturer's instructions. For bulk-phase measurements, the electrode was directly immersed in the corresponding biofilm supernatant. For intrabiofilm measurements, the microelectrode tip was carefully positioned within the interior of the intact or DNase-treated biofilm, and the signal was recorded after stabilization.

**H_2_S-induced bacterial persistence analysis.** To investigate whether H_2_S induces a persister-like state in bacteria, *S. aureus* was treated with different concentrations of Na_2_S prior to subsequent analyses.

Bacteria exposed to different concentrations of Na_2_S were collected, and intracellular ATP levels were determined using the BacTiter-Glo Microbial Cell Viability Assay kit (Promega) according to the manufacturer's instructions.

The fluorescence dilution assay was performed as previously described [35]. Briefly, *S. aureus* were pre-labeled with CFSE (Catalog No. S8269, Selleckchem, Houston, TX) and then cultured in LB medium containing different concentrations of Na_2_S. After 16 h of incubation, bacteria were harvested from the culture medium and analyzed by flow cytometry. Fluorescence dilution was used to assess bacterial proliferation, with retention of stronger CFSE fluorescence indicating reduced cell division.

The TDtest assay was carried out according to the procedures from previous studies [47]. The overall TDtest consisted of two main steps. Step I: Approximately 10^6^-10^7^ bacteria pretreated by different formulation were plated evenly on LB agar plates. Then, a sterile paper disk containing Cip was added to the center of the plate. The plates were incubated at 37 °C for 18 h. Step II: The antibiotic paper disk was replaced with a sterile paper disk containing 10 μL of 40% glucose, and the plates were further incubated at 37 °C overnight.

To determine whether H_2_S reduced bacterial susceptibility to hydrogen peroxide, Na_2_S-pretreated and untreated *S. aureus* were spread onto LB agar plates. A sterile susceptibility disk soaked with H_2_O_2_ was placed at the center of each plate. After incubation at 37 °C for 24 h, the diameter of the inhibition zone surrounding the disk was measured and compared between groups.

**H_2_S-induced Immunosuppression analysis.** RAW264.7 was co-incubated with different concentration of Na_2_S at 37 °C for 12 h. Intracellular RNS were determined by an RNS detection kit (BBoxiProbe®O52, BestBio). After 12 h of different treatments, cells were washed by PBS and incubate RNS probe for 30 min at 37 °C. After washing with PBS buffer, fluorescence detection was performed in the FITC Channel under flow cytometry or CLSM. To detect the intracellular expression level of iNOS, after incubation for 12 h, the RAW264.7 cells were fixed with 4% paraformaldehyde, permeabilized with 0.1% Triton-X, and blocked by 1% bovine serum albumin (BSA). After washing, the cells were incubated with primary monoclonal antibodies for iNOS (1:100, Bioss-0162R) at 4 °C for 12 h. After primary antibodies were removed, the secondary antibodies (ProteinFind, HS111-01) were added at 1:200 and incubated for another 2 h. Subsequently, cells were stained by rhodamine phalloidin (Beijing Solarbio Science & Technology Co., Ltd., CA1610) and DAPI. Finally, the cells were observed and imaged with CLSM.

**Assessment of H_2_S protection against H_2_O_2_-mediated eDNA degradation.** eDNA was extracted from mature *S. aureus* biofilms by two cycles of vortexing for 5 min and sonication for 10 min in 1 mL sterile deionized water. Bacterial cells were removed by centrifugation at 5000 × g for 10 min at 4 °C, and the resulting supernatant containing extracellular polymeric substances was collected and kept on ice. The supernatant was then divided into four equal aliquots and subjected to the following treatments: PBS, Na_2_S, H_2_O_2_, or Na_2_S pretreatment followed by H_2_O_2_ exposure. In the sequential-treatment group, the extracted eDNA was first incubated with Na_2_S at a final concentration of 400 μM and subsequently exposed to H_2_O_2_ at a final concentration of 1 mM for 24 h. The corresponding single-treatment groups received Na_2_S or H_2_O_2_ alone, while PBS served as the control. After treatment, residual eDNA concentrations were quantified using a NanoDrop™ 2000c spectrophotometer and normalized to the PBS-treated control group.

**Synthesis of FC decorated by azide.** 50 mg FeSt_3_ and 20 mg Cip was dissolved in 10 mL propan-2-ol (called Solution A). In the water bath at 50 °C, 15 mg DSPE-PEG_2000_-N_3_ and 45 mg L-α-Lecithin were added to 40 mL of 4% aqueous solution of ethanol and stirred at 1500 rpm for 30 min (called Solution B). Then, 3 mL of Solution A was added dropwise to Solution B. The mixture was kept in a water bath at 50 °C, and stirred at 1500 rpm for 1 h. Then mixture was harvested and washed three times by PBS to remove excess azide groups. FeSt_3_ nanoparticles (FeSt_3_ NPs) were made by the same method in the absence of Cip. We labeled red fluorescence to FeSt_3_ NPs through the introduction Nile red during synthesis.

The drug loading content (DLC) of Cip in the FeSt_3_ and FeSt_3_/Cip (FC) formulations was quantified using UV-Vis spectrophotometry. The measurement was based on Cip's characteristic absorbance at a wavelength of 315 nm. The protocol involved the following steps: ①Establishment of a Standard Curve: A standard calibration curve was generated by measuring the absorbance of a series of Cip reference solutions at various known concentrations; ②Measurement of Unencapsulated Cip: The FC formulation was centrifuged, and the absorbance of the resulting supernatant was measured to determine the concentration of free, unencapsulated Cip; ③Calculation of DLC: DLC (%) = [(Total mass of Cip − Mass of free Cip)/Total mass of Cip] × 100%.

**Characterization of FC nanoparticles and their H_2_S-responsive behavior.** The morphology of FC NPs was examined by transmission electron microscopy (TEM). To evaluate H_2_S-responsive structural disassembly, FC NPs were incubated with Na_2_S and subsequently analyzed by TEM and CLSM. Nile Red was incorporated into the nanoparticles during preparation to visualize particle-associated fluorescence by CLSM.

The hydrodynamic diameter and polydispersity index (PDI) of FC NPs were measured using a Brookhaven 90Plus PALS instrument. Samples were diluted 1:1000 in deionized water and measured at 25 °C. To assess batch-to-batch reproducibility, three independently prepared batches of FC NPs were analyzed before and after incubation with 400 μM Na_2_S. Each batch was measured in triplicate, and the Z-average diameter and PDI were recorded.

The chemical changes associated with the H_2_S-responsive reaction were further characterized by X-ray photoelectron spectroscopy (XPS). FC NPs before and after Na_2_S treatment were collected and analyzed by survey XPS and high-resolution Fe 2p spectra to evaluate sulfur incorporation and changes in the Fe(III)/Fe(II) composition. H_2_S depletion by FeSt_3_-containing formulations was evaluated using an H_2_S assay kit (Solarbio, Beijing, China) and lead acetate test strips (Aladdin Biochemical Technology Co., Shanghai, China).

**Minicell^P2O^ preparation from *EcN* using genetic engineering.** Deletion of the *minCD* gene was carried out by Hangzhou Biosci Biotech Co., Ltd. The pGEX-4T-P2O-T2A-EGFP plasmids were synthesized by Hunan Fenghui Biotechnology Co., Ltd. The pGEX-4T-P2O-T2A-EGFP plasmids were electroporated into Δ*EcN* cells and screened on an ampicillin-containing LB plate to obtain the positive strain (Δ*EcN* harboring the pGEX-4T-P2O-T2A-EGFP plasmid) following the general protocol.

To purify minicell from parent bacterial cells, culture of Δ*EcN* cells harboring the pGEX-4T-P2O-T2A-EGFP plasmid was subjected to initial differential centrifugation at 2000 g for 10 min to decrease the bulk of the bacterial burden. The resulting bacterial cell/minicell supernatant was subjected to centrifugation at 12000*g* for 10min, and the precipitate was collected as minicell.

**Synthesis of Minicell^P2O^ decorated by DBCO.** The minicell purified from Δ*EcN* harboring the pGEX-4T-P2O-T2A-EGFP plasmid (minicell^P2O^) were washed with PBS twice and then suspended in PBS. DBCO-PEG_2000_-NHS was then added into the suspension to a final concentration of 100 μM. After incubation for 4 h, the bacteria were centrifuged at a speed of 12000g for 10 min and then resuspended in PBS. The minicell was made by the same method in the absence of pGEX-4T-P2O-T2A-EGFP plasmid.

**Synthesis of MPFC.** The azido-decorated FC NPs and DBCO-decorated minicell^P2O^ (GFP-labeled) were resuspended in PBS and mixed in a 1:1 ratio. The mixture was coincubated for another 4 h to obtain MPFC. Minicell^P2O^@FeSt_3_ (MPF) was made by the same method in the absence of Cip. Minicell@FeSt_3_/Cip (MFC) was made by the same method in the absence of plasmid.

**Characterization of MPFC assembly.** Formation of the MPFC biohybrid was characterized by Fourier-transform infrared (FTIR) spectroscopy, TEM, dynamic light scattering (DLS), and CLSM. FTIR spectra of FC NPs, minicell^P2O^, and MPFC were recorded to verify the integration of the nanoparticle and minicell components. The morphology of MPFC and the attachment of FC NPs to the minicell surface were directly examined by TEM.

To further evaluate the predominant 1:1 conjugation pattern, FC NPs of different sizes were conjugated with minicells under otherwise identical conditions and examined by TEM. The hydrodynamic diameters of FC NPs, minicell^P2O^, and MPFC were measured by DLS as described above. For fluorescence imaging, Nile Red-labeled FC NPs and GFP-expressing minicells were visualized by CLSM to confirm their spatial association. Expression of the GST-P_2_O fusion protein in minicell^P2O^ and MPFC was confirmed by Western blotting using an anti-GST antibody (Proteintech, 66001-2). The cytocompatibility of MPFC was evaluated using a Cell Counting Kit-8 assay.

**Intrinsic antibacterial and antibiofilm activity of minicells.** The effects of parental *EcN* and *EcN*-derived minicells on *S. aureus* were evaluated using planktonic growth, biofilm-formation, and preformed-biofilm assays. Bacterial growth was monitored over 24 h by measuring OD_600_. For biofilm assays, *S. aureus* was cultured with the indicated treatments either during biofilm formation or after mature biofilms had formed. Biofilm biomass was quantified by 0.1% crystal violet staining for 30 min, followed by dissolution in 1 mL ethanol and absorbance measurement at 575 nm.

**Anaerobic migration.** Anaerobic migration of MPFC was investigated using a Transwell system (polycarbonate membrane: 3 μm pore size, 6.5 mm diameter, and 0.33 cm^2^ membrane surface area; Corning, USA). MPFC (200 μL), together with equivalent concentrations of minicells or FC NPs, was added to the upper chamber. To establish hypoxic conditions, the lower chamber contained 0.4 mL glucose solution (0.4 mg/mL), glucose oxidase (0.5 KU), and catalase (0.5 KU), whereas 0.4 mL glucose solution alone was used for the normoxic condition. Oxygen levels were assessed using the oxygen-sensitive probe tris (4,7-diphenyl-1,10-phenanthroline)ruthenium(II) chloride ([Ru(dpp)_3_]Cl_2_; Maokang Biotechnology Co., Ltd., China). To further verify the vertical oxygen gradient, samples from the upper and lower chambers were collected under the same conditions and their probe fluorescence was measured using a microplate reader. To distinguish active migration from passive transport, minicells were fixed with 4% paraformaldehyde, thoroughly washed, and subjected to the same hypoxic Transwell assay as a nonmotile control. GFP-labeled minicells recovered from the lower chamber were quantified by flow cytometry, while Nile Red-labeled FC or MPFC was detected using the IVIS system.

**Observation of Penetration about MPFC in biofilm.** The penetration of different formulation to biofilm was detected as previously described. Colonies of *S. aureus* were grown on top of filter membranes. Then, FITC-ConA was used to label the biofilm in *vitro* (FITC-biofilm). Then we labeled Nile red to FeSt_3_ NPs through the introduction Nile red during FeSt_3_ NPs synthesis. Non-deformable red fluorescent PS microsphere (100-500 nm) were bought from Tianjin BaseLine ChromTech Research Centre. The pBV220-mCherry plasmid was electroporated into Δ*EcN* cells to obtain minicell^mCherry^. The minicell@PS (MPS) was obtained by coincubating azido-decorated PS and DBCO-decorated minicells. Different formulations were co-cultured with FITC-biofilm for 30 min, 60 min and 90 min, the sliced biofilm by frozen slicer (Leica CM1900, Germany) and placed on glass slides for CLSM observation. The depth of penetration and area of penetration were calculated by ImageJ. For three-dimensional analysis, intact biofilms treated with PS, FeSt_3_, MPS, or MF were additionally imaged by CLSM using serial z-stack scanning from the biofilm surface to the substratum, followed by three-dimensional reconstruction to visualize the vertical distribution of particle-associated fluorescence throughout the biofilm.

**Detection the ROS level in minicells.** Intracellular ROS were determined by dichlorofluorescein diacetate staining (DCFH-DA, Solarbio, Beijing, China). The minicells cultured for different times and *S. aureus* biofilm after different treatments were washed with PBS and co-cultured with 1:1000 dilution of DCFH-DA at 37 °C for 30 min. After washing with PBS again, fluorescence detection was performed using the FITC channel of the flow cytometer and CLSM.

**Detection of H_2_O_2_ Concentration.** Minicell, MP and MPFC were collected and resuspended in 1 mL PBS, addition of 200 μL 5 mg/mL glucose solution, and incubation at 37 °C for different time. Then, the supernatant was collected. The sample interacted with 200 μL of 1 mg/mL ABTS (2, 2′-azino-bis(3-ethylbenzothiazoline-6-sulfonic acid)) and 200 μL of 1 mg/mL horseradish peroxidase for 12 h at 4 °C. This could prove the presence of H_2_O_2_ if the sample turned blue, and detect the change in absorbance at 420 nm.

**Validation of Fe^2+^-Mediated Catalytic ROS Generation via Iron Chelation.** To definitively confirm that the observed Fenton-like ROS generation was catalyzed by ferrous ions (Fe^2+^) released from the FeSt_3_ after reacting with H_2_S, an iron chelation experiment was performed using ethylenediaminetetraacetic acid (EDTA). First, the FeSt_3_ were “activated” by incubation with a NaHS solution (to mimic the H_2_S-rich biofilm microenvironment and trigger the formation of metastable iron sulfides, which serve as the Fe^2+^ source). Following this activation step, the system was divided into two groups. To the experimental group, EDTA was added to a final concentration of 400 μM to sequester any released Fe^2+^ ions. The control group received an equal volume of PBS instead of the EDTA solution. Subsequently, the generation of ROS in both systems was quantified using the DCFH-DA probe, as previously described. The fluorescence intensity of the EDTA-treated group was compared to that of the control group to assess the degree of ROS suppression and thereby validate the critical role of free ferrous ions in the catalytic process.

**Dispersion of biofilm experiments in *vitro*.** To detect the biofilm dispersing property of the nanocomplex, titanium plates were soaked in LB with *S. aureus* in 24-well plates, placed at 37 °C, and incubated for 4 days to construct biofilm modules. Then, the biofilms treated with different NPs for 12 h were subjected to biofilm assays. Firstly, some biofilms were fixed in 4% paraformaldehyde, and stained with PI (Beyotime, China) to observe the eDNA in biofilm by CLSM. Some of biofilms were fixed in 4% paraformaldehyde, dehydrated in gradient alcohol, and observed by SEM. FTIR was used to determine the types of functional groups contained in EPS. Biofilms treated by different NPs were scraped off the carrier surfaces and air dried. The dried samples were measured in ATR-FTIR mode between 400 and 4000 cm^−1^. The biofilm storage modulus (G′, elasticity modulus) and loss modulus (viscous modulus, G″) were determined by shear rheometry. Differently treated *S. aureus* biofilm were scratched and transferred immediately to the rheometer. Oscillatory frequency sweeps from 0.1 to 600 rad/s at 1% strain were performed on each sample at 37 °C.

To analyze the breakage of eDNA by ROS and quantification of eDNA in the biofilm matrix treated by different NPs, *S. aureus* biofilm in different groups were collected as described above. The concentration of extracted eDNA was quantified using the NanoDrop™ 2000c Spectrophotometer.

Some tablets were transferred to a sterile 15 mL centrifuge tube with 1 mL PBS, respectively, followed by ultrasonic oscillation for 10 min and vortexed mixture for 2 min to dislodge the biofilm. Afterwards, the suspensions were centrifuged to extract *S. aureus*, and resuspended in PBS. The cellular ROS generation in *S. aureus* cells was characterized using DCFH-DA indicator. Then, it was slightly washed three times with sterile PBS. At last, the fluorescence images of DCF were visualized by employing CLSM and flow cytometry.

**Antibiofilm experiments in *vitro*.** The biofilm was modeled and treated differently as described above. To detect the antibiofilm property of the NPs, after being treated by different groups, the biofilms cultured on titanium plates were stained with 0.1% crystal violet for 30 min. Then, stained biofilms were gently washed with PBS twice. To quantify the biomass of the biofilms, crystal violet were dissolved into 1 mL ethanol and the absorbance at 575 nm was recorded with a microplate reader. A portion of the biofilms was stained with the Live/Dead Bacterial Staining Kit (BestBio-4126) and then observed with CLSM.

Some tablets were transferred to a sterile 15 mL centrifuge tube with 1 mL PBS, respectively, followed by ultrasonic oscillation and vortexed mixture to dislodge the biofilm as described before. And then, a part of suspensions were serially diluted and spread on the LB agar plate, which was further incubated at 37 °C overnight to count the colony-forming units(CFU).

**Antibacteria mechanism and persister assays.** Bacteria from biofilms were treated for 8 h with different NPs. At specified time points, bacteria were removed, serially diluted and spot-plated onto LB agar plates to determine CFU/ml. Survival rate was determined. The MICs of different formulations against bacteria from biofilm were tested separately at 37 °C. Bacteria after treatment at concentrations above MIC were diluted onto LB agar plates to detect the MBC.

Fluorescence dilution experiments were performed as previously described. Briefly, *S. aureus* prestained by CFSE (S8269, Selleck) were grown in LB. After 16 h culturing, bacteria were extracted from LB analyzed on a flow cytometer.

Bacteria were cultured with different concentrations of MPFC. The ATP level of each sample was measured using the BacTiter Glo Kit (Promega) according to the manufacturer's instructions. Bacteria with different treatment incubated with H_2_S fluorescent probe WSP-1 for 30 min at 37 °C away from light. After washing by PBS, the fluorescent intensity was measured by flow cytometer.

Time-kill curve analysis was performed as previously described. The number of CFUs was then counted. And bacteria pretreated with different formulations were further subjected to the TDtest and hydrogen peroxide disk diffusion assay to evaluate their drug susceptibility.

**Temporal-order Perturbation Assay.** To evaluate the functional importance of the treatment sequence, biofilm-derived *S. aureus* were sequentially treated with PBS-PBS, FeSt_3_-PBS, PBS-H_2_O_2_, H_2_O_2_-FeSt_3_, or FeSt_3_-H_2_O_2_. The FeSt_3_ and H_2_O_2_ doses and total treatment duration were kept identical between the two sequential-treatment groups. After treatment, bacteria were serially diluted and plated on LB agar, and bacterial survival was quantified as CFU/mL. All experiments were independently repeated three times.

**Immunomodulatory Experiments In *Vitro*.** We stimulated infection microenvironment via incubating macrophages with biofilms treated with different formulations in Transwell system. And then, the RAW264.7 cells in upper chamber were collected to detect the H_2_S levels by WSP-1. A part of cells were collected to detect the RNS and iNOS levels as described above.

**Assessment of Proinflammatory Cytokine Expression in Macrophages Treated with minicells and MPFC.** To evaluate the immunogenicity and inflammatory potential of minicells and the MPFC nanosystem, RAW264.7 macrophages were incubated with different treatments and the expression levels of IL-1β, IL-6, and TNF-α were determined by enzyme-linked immunosorbent assay (ELISA) kit (Proteintech Group, inc. #KE10003, #KE10002 and KE10007) according to the manufacturer's instructions. Cells were divided into three groups: Untreated control, minicell treatment (minicells, 1 × 10^7^/mL, 12 h), MPFC treatment (equivalent number of MPFC, 12 h).

**RNA-Seq Sample Preparation and Data Analysis.** RAW264.7 cells coincubated with different groups of biofilm for 12 h were collected and washed with PBS for 3 times. 1 mL of Trizol (Invitrogen) was added to extract RNA, then frozen in liquid nitrogen immediately. The samples were then sequenced with Illumina NovaSeq 6000 in Novogene Co, Ltd (Beijing, China). The analyses were carried out by Beijing Novogene Bio Technology Co., Ltd (Beijing, China).

**Validation of NF-κB-HIF-1α Axis Activation.** After respective treatments, cells were harvested and lysed in RIPA buffer (New Cell & Molecular Biotech, WB3100) supplemented with protease inhibitors. Equal amounts of total protein (20 μg per sample) were separated by SDS-PAGE (DG102, TransGen Biotech, China) and transferred onto PVDF membranes. After blocking with 5% non-fat milk, membranes were incubated overnight at 4 °C with primary antibodies against NF-κB, HIF-1α and β-actin (EMAR, EM31011-02, as a loading control). After incubation with HRP-conjugated secondary antibodies (1:5000 dilution), protein bands were visualized using an ECL chemiluminescence detection kit (P10100, New Cell & Molecular Biotech). Anti-NF-κB: From ImmunoWay Biotechnology (Cat. No.:YT3108) and Selleck (Cat. No.:F0006); Anti-HIF-1α (Cat. No.: 80933-1-RR): From Proteintech.

**SiRNA-Mediated Knockdown of HIF-1α in RAW264.7 Macrophages.** To assess the role of HIF-1α in MPFC-induced metabolic and immune reprogramming, targeted knockdown of HIF-1α was performed in RAW264.7 cells using specific siRNAs. RAW264.7 cells were seeded in 6-well plates at 2 × 10^6^ cells/well and cultured overnight. Cells were transfected with three HIF-1α-targeting siRNAs (Generay Biotech, Shanghai, China) or scrambled negative control siRNA using Lipofectamine 3000 reagent (Invitrogen, USA). After 48 h of transfection, cells were lysed for protein extraction. HIF-1α knockdown efficiency was verified by Western blot using a specific anti-HIF-1α antibody (Proteintech, 80933-1-RR, 1:1000 dilution). Then, cells were treated with different-treated biofilms for another 12 h as previously described. After treatments, the levels of inflammatory cytokines (IL-1β, IL-6, and TNF-α) in the cell supernatants were determined using commercial ELISA kits following the manufacturer's instructions (brought from Proteintech, Cat No. KE10003; KE10007; and KE10002).

**HIF-1α Rescue Experiment.** To further verify the specific role of HIF-1α, a genetic rescue experiment was performed after HIF-1α knockdown. RAW264.7 macrophages were first transfected with HIF-1α-targeting siRNA as described above and subsequently transfected with an HIF-1α expression plasmid encoding mouse *Hif1a* (NM_001313919.1). Restoration of HIF-1α expression was confirmed by Western blotting. The cells were then exposed to the indicated biofilm treatments for 12 h, and the concentrations of IL-1β, IL-6, and TNF-α in the culture supernatants were measured by ELISA as described above.

**Quasi-Targeted Metabolomics Sample Preparation and Data Analysis.** The RAW264.7 cells co-incubated with different groups of biofilm for 12 h were placed in the EP tubes and resuspended with prechilled 80% methanol by vortexed thoroughly. Then the samples were melted on ice and whirled for 30 s. After the sonification for 6 min, they were centrifuged at 5000 rpm, 4 °C for 1 min. The supernatant was freeze-dried and dissolved with 10% methanol. Finally, the solution was injected into the LC-MS/MS system for analysis. The analyses were carried out by Beijing Novogene Bio Technology Co., Ltd (Beijing, China).

**Intracellular lactate assay following HIF-1α knockdown.** RAW264.7 macrophages were subjected to HIF-1α knockdown as described above, with parallel cultures without knockdown serving as controls. Cells were subsequently exposed for 12 h to untreated biofilms or biofilms treated with Cip, FC, or MPFC. After treatment, cells were washed with PBS and lysed with BeyoLysis™ Buffer A for Metabolic Assay. Cell lysates were incubated on ice for 10 min and centrifuged at 12,000 × g for 5 min at 4 °C. Intracellular L-lactate concentrations in the resulting supernatants were determined using an L-Lactate Assay Kit with WST-8 (Beyotime Biotechnology, Shanghai, China; Cat. No. S0208S) according to the manufacturer's instructions. Absorbance was measured at 450 nm, and L-lactate concentrations were calculated from the corresponding standard curve.

**Assessment of metabolic-Dependent Immune Activation Using 2-Deoxy-D-Glucose (2-DG) Inhibition Assay.** To investigate the causal link between metabolic reprogramming and immune activation in MPFC-treated macrophages, we performed glycolysis inhibition experiments using 2-deoxy-D-glucose (2-DG). RAW264.7 macrophages were seeded in plates and pretreated with 2-DG (10 mM, Macklin) for 4 h at 37 °C to inhibit glycolysis. After pretreatment, cells were stimulated with different-treated biofilms for 12 h. Following stimulation, the expression levels of IL-1β, IL-6, and TNF-α were analyzed by ELISA as previously described. All experiments were performed in triplicate.

**Mitochondrial ROS Assays.** Mito-SOX red mitochondrial superoxide indicator (Yeasen Biotech, #40778ES50) was used to analyze mitochondrial ROS. RAW264.7 with different treatments were then co-incubated with Mito-SOX in the dark for 10 min at 37 °C. And then cells were washed by PBS, and stained by DAPI. The RAW264.7 cells were observed with CLSM.

**ATP assays in cells.** RAW264.7 were cultured with different concentrations of Na_2_S or biofilm treated with different formulations. The ATP level of each sample was measured using the BacTiter Glo Kit (Promega) and ATP content test kit (BC0300, Beijing Solarbio Science & Technology Co., Ltd.) according to the manufacturer's instructions.

***In situ* implant infection model.** Male BALB/c mice (6-8 weeks old) underwent subcutaneous titanium plate implantation. Briefly, the mice were anesthetized, shaved, and disinfected, and the dorsal skin was incised under sterile conditions to expose the subcutaneous layer. Sterile titanium plates (diameter, 5 mm) were implanted subcutaneously, followed by wound closure. Subsequently, 100 μL of *S. aureus* suspension (1 × 10^6^ CFU/mL) was inoculated onto each implant. Three days after infection, mice were randomly assigned to the indicated treatment groups, including PBS, Cip, FC, and MPFC, and 100 μL of the corresponding formulation was administered to the infection site. Independent animals were allocated to different experimental endpoints, including bacterial burden assessment, histological analysis, TDtest, local tissue flow cytometry, lymph-node immune profiling, and *in vivo* targeting experiments. The number of biologically independent animals used for each endpoint is indicated in the corresponding figure legends.

Seven days after treatment, animals allocated to bacterial burden analysis were euthanized, and the implants and surrounding tissues were aseptically collected and kept on ice. Peri-implant tissues were weighed and homogenized using a tissue homogenizer. Implant-associated bacteria were recovered by three cycles of sonication for 10 min followed by vortexing for 30 s. The resulting bacterial suspensions were serially diluted with PBS, plated on LB agar, and incubated at 37 °C overnight. Bacterial counts were expressed as CFU per implant or normalized to tissue weight as CFU/g. Implants collected for morphological analysis were processed for SEM as described for the *in vitro* biofilm experiments. Peri-implant tissues allocated to histological analysis were fixed in 4% paraformaldehyde and processed for H&E, Masson's trichrome, and immunofluorescence staining.

To further evaluate bacterial persistence *in vivo*, viable bacteria recovered from peri-implant tissues after the indicated treatments were subjected to the TDtest as described above.

For quantitative characterization of the local immune microenvironment, peri-implant tissues from independently allocated animals were collected and processed into single-cell suspensions. Tissues were minced and enzymatically dissociated using Primary Tissue Digestion Solution (JFKR-Organoid, Cat. No. JFKR-ZXHY-100) according to the manufacturer's instructions. The resulting suspensions were filtered through a 70-μm cell strainer and treated with ACK lysis buffer to remove erythrocytes. Fc receptors were blocked with anti-CD16/32 antibody before surface staining. Cells were stained with fluorescent antibodies against CD45, CD11b, F4/80, CD86, and CD206. Macrophages were identified as CD45^+^CD11b^+^F4/80^+^ cells. The proportion of macrophages among CD45^+^ immune cells, the percentages of CD86^+^ and CD206^+^ cells within the macrophage population, and the CD86^+^/CD206^+^ ratio were subsequently quantified by flow cytometry. Antibody information and dilutions are listed in Supplementary Table S2.

For immune profiling of the draining lymph nodes, inguinal and axillary lymph nodes were harvested immediately after euthanasia and mechanically dissected into small pieces. The tissues were digested with 1 mg/mL collagenase IV and 100 μg/mL DNase at 37 °C for 30 min, filtered through a 70-μm cell strainer, and treated with ACK lysis buffer (Gibco) for 5 min at room temperature. After Fc receptor blockade with anti-CD16/32 antibody for 10 min, cells were stained on ice for 30 min with fluorescently labeled antibodies against CD80 (16–10A1), CD11c (N418), I-A/I-E (M5/114.15.2), CD86 (GL-1), CD3 (17A2), CD8a (53–6.7), CD4 (GK1.5), CD19 (6D5), and CD138 (281-2). For intracellular Foxp3 staining, cells were fixed and permeabilized using the FoxP3/Transcription Factor Staining Buffer Kit (IC001, Multisciences), followed by staining with anti-Foxp3 antibody (MF-14). Samples were acquired using a NovoCyte Quanteon flow cytometer and analyzed using NovoExpress. Detailed gating strategies are provided in the Supplementary Information, and antibody information and dilutions are listed in Supplementary Table S2.

For the *in vivo* biofilm-targeting assay, *S. aureus* biofilms formed on titanium implants were first labeled with FITC-ConA (5 μg/mL in PBS) for 15 min at 37 °C in the dark and gently washed with PBS to remove excess dye. The FITC-ConA-labeled biofilm-bearing implants were then implanted subcutaneously into mice. Nile Red-labeled FC or MPFC was subsequently administered at a site distal to the implant. After 24 h, the mice were euthanized, and the titanium implants were aseptically retrieved and immediately examined by CLSM to assess the accumulation of Nile Red-labeled FC or MPFC at the FITC-ConA-labeled biofilm site.

***In vitro* dendritic cell maturation assay.** The bone-marrow-derived DCs (BMDCs) were seeded in the lower chamber of the Transwell system, whereas RAW264.7 macrophages subjected to the indicated treatments were placed in the upper chamber and cocultured with BMDCs for 24 h. Then, the bottom DCs were observed by microscope. Subsequently, the single-cell suspension was incubated with anti-CD16/32 antibody for 10 min to block Fc receptors, and further incubated with fluorescent-labeled anti-mouse antibodies against CD80, CD11c, and CD86 for 30 min on the ice. Multiparameter analyses were performed on flow cytometer, and analyzed using flowjo. Antibody information and dilutions are listed in Supplementary Table S3.

**Recurrent implant infection model *in vivo*.** To establish a recurrent implant infection model, male BALB/c mice (Six-to-eight-week-old, n = 24) were given sterile titanium plates (Φ 5 mm) and an inoculation of *S. aureus* (100 μL, 10^6^ CFU/mL) on day 1. 3 days later, mice were randomly divided into four groups: PBS (PBS-treated group), Cip, FC, and MPFC. The infected site was injected with 100 μL of the specified formulation. Under anesthesia, the surgical region of each mouse was disinfected with skin on day 10. The titanium plates were taken out, and the skin was excised sterilely. The wound was sutured once the diseased tissues had been debrided and removed. The following day, new sterile titanium plates were put into the previously operated subcutaneous region and then re-injected with fresh *S. aureus* (100 μL, 10^6^ CFU/mL). The surgical area was photographed daily before the mice were euthanized on day 24. The removed titanium plates were placed in 1 mL of PBS for sonication and vortexing, and the bacterial suspensions were diluted with PBS, plated on LB agar plate, cultured at 37 °C overnight. Some of the tissues around the tablets were removed and fixed in 4% paraformaldehyde to cut into ultrathin sections for staining. Inguinal and axillary lymph nodes were promptly collected and treated as described above to obtain the single-cell suspension. After Fc receptor block with anti-CD16/32 antibody, the cells were incubated with antibodies against B220 (RA3-6B2), IgD (11-26 c.2a), IgG (Poly4053) and CD80 (16–10A1). The information for staining antibodies and dilutions is provided in Supplementary Table S4. Samples were then acquired with the flow cytometer. At the experimental endpoint, blood samples were collected and processed to obtain serum. Total serum IgG levels were measured using a Mouse IgG ELISA Kit (Cat. No. ADS-W-D036, Jiangsu Aidisheng Biological Technology Co.,Ltd) according to the manufacturer's instructions, as previously reported [42].

**Intraosseous implant infection model *in vivo*.** Female SD rats (200-220 g, n = 60) were used. After anesthesia, a cortical bone defect (approximately 6 mm × 1 mm) was created in the proximal tibia, followed by implantation of a sterile titanium rod (Φ 1 mm × 6 mm). Except for the Sham and MPFC(−) groups, 100 μL of *S. aureus* suspension (10^6^ CFU/mL) was inoculated into the defect.

Rats were assigned to nine groups: PBS, Cip, minicells, FeSt_3_, FC, MPF, MPFC, Sham, and MPFC(−). The MPFC(−) group consisted of three rats and served as an uninfected safety control. Each of the other eight groups included six rats, with three used for histological analysis and three for tissue homogenization and bacterial burden assessment (48 rats). In addition, three extra rats each in the PBS, Cip, and MPFC groups were used for micro-CT analysis (9 rats), giving a total of 60 rats.

Three days after model establishment, the corresponding treatments were administered. One month later, the rats were euthanized and the tibiae were harvested. Micro-CT was performed in the PBS, Cip, and MPFC groups (n = 3 per group). Major organs were collected from all groups and subjected to H&E staining for histological safety evaluation.

## Data availability

The RNA-seq data have been deposited in the Sequence Read Archive (SRA) under accession number PRJNA1288601. The processed metabolomics dataset, including metabolite annotations, sample-level intensity values and sample information, is available in Zenodo under DOI 10.5281/zenodo.21757132.

## Statistical analysis

Statistical analyses were performed using GraphPad Prism. Data are presented as mean ± standard deviation (SD). Variance homogeneity was evaluated using the Brown–Forsythe test. Ordinary one-way ANOVA followed by Tukey's multiple-comparisons test was used for datasets satisfying the homogeneity-of-variance assumption, whereas Welch's one-way ANOVA with an appropriate multiple-comparisons procedure was used where variance heterogeneity was present. P < 0.05 was considered statistically significant. Significance levels are indicated as *P < 0.05, **P < 0.01, ***P < 0.001, ****P < 0.0001; ns, not significant.

## CRediT authorship contribution statement

**Mengying Yin:** Visualization, Validation, Methodology, Formal analysis, Data curation, Conceptualization. **Mingyue Su:** Methodology. **Jundan Yi:** Methodology. **Yanrong Ren:** Methodology. **Yueting Zhang:** Methodology. **Qian Wu:** Methodology. **Chang Yang:** Methodology. **Shuya Xiao:** Methodology. **Cen Gao:** Methodology. **Rongbing Tang:** Methodology. **Bin Cheng:** Writing – review & editing, Supervision, Project administration, Funding acquisition. **Juan Xia:** Writing – review & editing, Supervision, Project administration, Funding acquisition.

## Ethics approval and consent to participate

All animal experimental protocols were performed strictly following the guidelines established by the Association of Laboratory Animal Sciences and the Center for Laboratory Animal Sciences at Lanzhou University (approval number: LZUKQ-2023-043, LZUKQ-2025-084 and LZUKQ-2026-114), the Center for Laboratory Animal Sciences at Sun Yat-sen University (approval number: SYSU-IACUC-2025-B1055).

## Funding sources

This study was supported by the 10.13039/501100001809National Natural Science Foundation of China (U23A20445, 82470995, 82101011), the Fundamental Research Funds for the Central Universities (lzujbky-2022-ey19), Gansu Association for Science and Technology (GXH20220530-16), Natural Science Foundation of Gansu Province of China (20YF8WA084, 20YF8FA073), Clinical Research Center for Oral Diseases, Gansu Province (20JR10FA670), the open fund of Key Laboratory of Dental Maxillofacial Reconstruction, Biological Intelligence Manufacturing, Gansu Province (20JR10RA653, ZDKF20210301) and Hospital of Stomatology Lanzhou University Scientific Research Project (lzukqky-2021-q01, lzukqky-2021-q07).

## Declaration of competing interest

The authors declare that they have no known competing financial interests or personal relationships that could have appeared to influence the work reported in this paper.

## Footnotes

Appendix A Supplementary data to this article can be found online at https://doi.org/10.1016/j.bioactmat.2026.08.049.
