# Supplementary material for: Dismantling the eDNA-mediated H2S barrier with biohybrids against refractory biofilm infections
Source: Bioact Mater. 2026 Sep 15;68:695–715. doi: 10.1016/j.bioactmat.2026.08.049 (PMC13598538; doi:10.1016/j.bioactmat.2026.08.049)
Supplement: Supplementary file 1 [file mmc1.docx]

**Supporting Information**

**Dismantling the eDNA-Mediated H_2_S Barrier with Biohybrids against Refractory Biofilm Infections**

Figure S1. eDNA contributes to H_2_S retention by the biofilm matrix. Equal amounts of intact or DNase-treated lyophilized biofilms were incubated with Na_2_S solution for 24 h, and the residual H_2_S concentration in the supernatant was measured using an H_2_S-selective microelectrode, indicating that removal of eDNA reduced the sulfide-retention capacity of the biofilm matrix.

Figure S2. Radial distribution analysis reveals preferential but weak association of H_2_S with the DNA phosphate backbone. Radial distribution functions (RDFs) of the sulfur atom of H_2_S relative to phosphorus atoms (S-P), phosphate oxygen atoms (S-OP), and nucleobase atoms (S-Base) of DNA. The RDF profiles indicate preferential localization of H_2_S near phosphate backbone atoms rather than nucleobase regions.

Figure S3. Quantification of H_2_S enrichment within biofilms before and after DNase treatment. H_2_S concentrations were measured in the biofilm supernatant, intact biofilm interior, and DNase-treated biofilm interior using an H_2_S-selective microelectrode.

Figure S4. H_2_S attenuates H_2_O_2_-mediated eDNA loss. Equal amounts of biofilm-derived eDNA were treated with Na_2_S, H_2_O_2_, or Na_2_S pretreatment followed by H_2_O_2_ exposure. Residual DNA concentrations were measured using a NanoDrop spectrophotometer and normalized to the mean value of the untreated control group, which was set to 100%.

Figure S5. Schematic illustration of the synthesis of MPFC.

Figure S6. Quantification of the Cip drug loading content (DLC). (a) Standard calibration curve of Cip measured by UV‒Vis spectrophotometry at 315 nm. (b) Absorption curves of the FeSt_3_ and FC supernatants after centrifugation.

Figure S7. Validation of H_2_S scavenging by FeSt_3_ and MPFC in a live bacterial culture. Both FeSt_3_ and MPFC markedly reduced the blackening of the lead acetate paper, indicating efficient removal of bacterially-produced H_2_S directly within the culture medium.

Figure S8. Plasmid map of pGEX-4T-P2O-T2A-EGFP showing key genetic elements including the GST tag for purification and detection, the P_2_O therapeutic gene, the T2A self-cleaving peptide, and the EGFP reporter gene for tracking.

Figure S9. CLSM image confirming GFP expression in minicell^P2O^. The strong green fluorescence indicates the successful expression and inheritance of the plasmid-encoded EGFP reporter protein within the purified minicells. Scale bar = 5 µm.

Figure S10. Experimental validation of steric hindrance as the determinant for 1:1 conjugation. TEM images show that when FC NPs are similar in size or larger than the minicell (left panel), a 1:1 conjugation stoichiometry is exclusively observed. In contrast, using significantly smaller FC NPs (middle panel) results in multiple nanoparticles attaching to a single minicell. A mixed-size conjugation experiment (right panel) further confirms this principle within the same sample.

Figure S11. Assessment of metabolic activity in minicells via FDA staining. The green fluorescence results from the hydrolysis of nonfluorescent fluorescein diacetate (FDA) into fluorescein by active endogenous esterases within the minicells, confirming their enzymatic viability. Scale bar = 20 µm.

Figure S12. Effect of *EcN* and minicells on *S. aureus* growth and biofilm formation. (a) Growth curves of *S. aureus* with different treatment over 24 hours. (b) Quantification of biofilm formation by crystal violet staining. (c) Quantification of biofilm dispersal by crystal violet staining.

Figure S13. CCK-8 quantification data for RAW264.7 macrophages subjected to different treatments for 24 h.

Figure S14. Validation of the hypoxic Transwell system and motility-dependent minicell migration. (a) Representative oxygen-sensitive fluorescence images of the lower chamber in the presence GOx(+) or absence GOx(−) of the GOx/catalase oxygen-scavenging system, detected using [Ru(dpp)_3_]Cl_2_. (b) Quantitative comparison of oxygen-sensitive fluorescence between the upper and lower chambers under hypoxic conditions. (c) Concentrations of live and paraformaldehyde-fixed minicells recovered from the lower chamber under hypoxic conditions. Statistical significance was assessed using a two-tailed paired Student’s t-test in (b) and a two-tailed Welch’s unpaired t-test in (c).

Figure S15. Three-dimensional distribution of particles within biofilms. Representative CLSM z-stack reconstructions of *S. aureus* biofilms treated with PS, FeSt_3_, MPS, or MF. Biofilms are shown in green, and particle-associated fluorescence is shown in red.

Figure S16. MPFC depletes H_2_S within the biofilm. (a) Representative fluorescence images of MPFC-treated biofilms stained with the H_2_S probe WSP-1. Scale bar = 50 μm. (b) Quantification of intrabiofilm H_2_S concentrations after PBS (as Control) or MPFC treatment using an H_2_S-selective microelectrode. Data are presented as mean ± SD (n = 3 paired independent biofilm samples). Statistical significance was determined using a two-tailed paired t-test.

Figure S17. Validation of Fe^2+^-mediated ROS generation via a Fenton-like reaction tested using an iron chelator. Flow cytometry showing intracellular ROS levels, measured by DCFH-DA staining and FITC fluorescence intensity, under different conditions: untreated control, H₂O₂ alone, H₂O₂ + EDTA, product (Fe sulfide) + H₂O₂, and product + H₂O₂ + EDTA. Compared with H₂O₂ alone, the addition of the product to H₂O₂ markedly increased ROS generation, indicating Fenton-like activity. Importantly, coincubation with the iron chelator EDTA substantially inhibited ROS production in both the H₂O₂ and product groups, confirming that the increase in ROS was mediated by free Fe^2+^ ions released from the product.

Figure S18. Effect of treatment order between H_2_S depletion and H_2_O_2_ exposure on bacterial survival. Biofilm-derived *S. aureus* were sequentially treated with PBS-PBS, FeSt_3_-PBS, PBS-H_2_O_2_, H_2_O_2_-FeSt_3_, or FeSt_3_-H_2_O_2_ under matched reagent doses and total exposure times. Bacterial survival was quantified by CFU plating.

Figure S19. Schematic diagram of macrophages within an *in vitro* simulated infection microenvironment in Transwell system.

Figure S20. Intrinsic immunogenicity of the therapeutic components in macrophages. RAW264.7 macrophages were incubated with minicells or MPFC in the absence of any biofilm-related stimulus. Untreated cells served as the negative control (Control). Secretion of the pro-inflammatory cytokines IL-1β, TNF-α, and IL-6 was quantified by ELISA.

Figure S21. Representative Western blot confirming the successful and significant knockdown of HIF-1α protein expression in siHIF-1α-transfected cells. A nontargeting siRNA (NC) was used as a negative control.

Figure S22. Knockdown of HIF-1α abrogates the MPFC-mediated upregulation of proinflammatory cytokines. **(a-c)** ELISA analysis showing the secretion levels of the proinflammatory cytokines IL-6 **(a)**, IL-1β **(b)**, and TNF-α **(c)** in the supernatants of siRNA-transfected macrophages.

Figure S23. Genetic rescue of HIF-1α in MPFC-treated macrophages. HIF-1α expression in RAW264.7 macrophages following HIF-1α knockdown and subsequent re-expression of siRNA-resistant HIF-1α. IL-1β, IL-6, and TNF-α levels in the indicated groups following MPFC treatment.

Figure S24. Intracellular lactate concentrations in RAW264.7 macrophages following the indicated treatments without (a) or with (b) HIF-1α knockdown.

Figure S25. Inhibition of glycolysis with 2-DG abrogates MPFC-mediated immunometabolic activation. Macrophages were pretreated with 2-DG, and the production of proinflammatory cytokines in the culture supernatants was determined by ELISA.

Figure S26. MitoSOX fluorescence probe was used to observe mitochondrial ROS activity after treatment with different concentrations of Na_2_S. (Scale bar = 100 μm)

Figure S27. *In vivo* accumulation of MPFC at infected biofilms. Biofilms were labeled with FITC-ConA (green), and FC or MPFC was labeled with Nile Red (red). After 24 h, implants were retrieved and imaged by CLSM. Scale bar = 50 μm.

Figure S28. MPFC reduces persister bacteria *in vivo*. Representative TDtest images of bacteria recovered from peri-implant tissues after different treatments. Late-emerging colonies were markedly reduced in the MPFC group, indicating depletion of the *in vivo* persister population.

Figure S29. Flow cytometric quantification of macrophage abundance and phenotypes in peri-implant tissues. Macrophages were identified as CD45^+^CD11b^+^F4/80^+^ cells. (a) Percentage of macrophages among CD45^+^ immune cells. (b-c) Percentages of CD86^+^ and CD206^+^ cells within the macrophage population. (d) CD86^+^/CD206^+^ ratio. Data are presented as mean ± SD (n = 3 biologically independent animals). Statistical significance was assessed by Welch’s one-way ANOVA followed by Dunnett’s T3 multiple-comparisons test.

Figure S30. Representative gating strategy for dendritic cells in the IDLN of the mouse *in situ* implant infection model.

Figure S31. MPFC restores dendritic cell maturation in a macrophage-DC coculture system. (a) Representative flow cytometry plots showing CD80 and CD86 expression in CD11c^+^ BMDCs after coculture with RAW264.7 macrophages subjected to the indicated biofilm-related treatments. (b) Quantification of CD80^+^CD86^+^ mature DCs among CD11c^+^ cells.

Figure S32. Representative gating strategy for CD4^+^ and CD8^+^ T cells in the IDLN of the mouse *in situ* implant infection model.

Figure S33. Representative gating strategy for plasma cells and plasmablasts in IDLN of the mouse *in situ* implant infection model.

Figure S34. Representative gating strategy for T_reg_ cells in IDLN of the mouse *in situ* implant infection model.

Figure S35. Representative images of H&E, Masson and Giemsa staining of tissues in the infected area of reinfection. Scale bar, 25 μm.

Figure S36. Representative gating strategy for memory B cells and CD80^+^ memory B cells in the IDLNs of the mouse model of recurrent implant infection.

Figure S37. Total serum IgG levels in the recurrent implant infection model. Serum samples were collected from mice following the indicated treatments, and total IgG concentrations were quantified by ELISA.

Figure S38. H&E-stained histological images of heart, liver, spleen, lung and kidney tissues harvested from rats in the intraosseous implant study (scale bar = 100 μm).

Table S1. Nomenclature and composition of the constructs used in this study.

| Construct | Composition | P_2_O plasmid | FeSt₃ | Cip |
| --- | --- | --- | --- | --- |
| FeSt_3_ | Iron(III) stearate nanoparticles | No | Yes | No |
| FC | FeSt_3_/Cip nanoparticles | No | Yes | Yes |
| minicell | Control minicells derived from *EcN*^Δ^*^minCD^* | No | No | No |
| minicell^P2O^/MP | P_2_O-expressing engineered minicells | Yes | No | No |
| MF | minicell@FeSt_3_ | No | Yes | No |
| MFC | minicell@FeSt_3_/Cip | No | Yes | Yes |
| MPF | minicell^P2O^@FeSt_3_ | Yes | Yes | No |
| MPFC | minicell^P2O^@FeSt_3_/Cip | Yes | Yes | Yes |
| MPS | minicell@polystyrene particles;  penetration control | No | No | No |

Table S2. Antibody list for Figure 8 and S29.

| **Colour** | **Antibody** | **Clone** | **Company (Cat.#)** | **Dilution** |
| --- | --- | --- | --- | --- |
| Alexa Fluor 700 | CD45.2 | 104 | Biolegend(109822) | 1:100 |
| FITC | CD11b | M1/70 | Biolegend(101206) | 1:100 |
| RB780 | CD86 | GL-1 | BD Biosciences(569220) | 1:100 |
| Alexa Fluor 647 | CD206 | C068C2 | Biolegend(141712) | 1:100 |
| PE | F4/80 | BM8 | Biolegend(123110) | 1:100 |
| PE | I-A/I-E | M5/114.15.2 | Biolegend (107607) | 1:100 |
| FITC | CD11c | N418 | Biolegend (117305) | 1:100 |
| APC | CD80 | 16–10A1 | Biolegend (104713) | 1:100 |
| PE/Cy7 | CD86 | GL-1 | Biolegend (105013) | 1:100 |
| FITC | CD3 | 17A2 | Biolegend (100203) | 1:100 |
| APC/Cy7 | CD8a | 53–6.7 | Biolegend (100714) | 1:100 |
| PerCP/Cy5.5 | CD4 | GK1.5 | Biolegend (100433) | 1:100 |
| APC | CD45 | 30-F11 | Biolegend (103111) | 1:100 |
| FITC | CD19 | 6D5 | Biolegend (115505) | 1:100 |
| PE | CD138 | 281-2 | Biolegend (142503) | 1:100 |
| PE | Foxp3 | MF-14 | Biolegend (126403) | 1:100 |

Table S3. Antibody list for Figure S31.

| **Colour** | **Antibody** | **Clone** | **Company (Cat.#)** | **Dilution** |
| --- | --- | --- | --- | --- |
| PE | CD86 | A17199A | Biolegend (159203) | 1:100 |
| FITC | CD11c | N418 | Biolegend (117305) | 1:100 |
| APC | CD80 | 16-10A1 | Biolegend (104713) | 1:100 |

Table S4. Antibody list for Figure 9.

| **Colour** | **Antibody** | **Clone** | **Company (Cat.#)** | **Dilution** |
| --- | --- | --- | --- | --- |
| APC | B220 | RA3-6B2 | Biolegend (103211) | 1:100 |
| PE | IgD | 11-26c.2a | Biolegend (405705) | 1:100 |
| FITC | IgG | Poly4053 | Biolegend (405305) | 1:100 |
| PE/Cy7 | CD80 | 16–10A1 | Biolegend (104733) | 1:100 |
